# Development of a 12-Week Unsupervised Online Tai Chi Program for People With Hip and Knee Osteoarthritis: Mixed Methods Study

**DOI:** 10.2196/55322

**Published:** 2024-09-30

**Authors:** Shiyi Julia Zhu, Kim L Bennell, Rana S Hinman, Jenny Harrison, Alexander J Kimp, Rachel K Nelligan

**Affiliations:** 1 Department of Physiotherapy, School of Health Sciences Centre for Health, Exercise and Sports Medicine University of Melbourne Melbourne Australia; 2 Rising Moon Tai Chi School Melbourne Australia

**Keywords:** intervention development, osteoarthritis, Tai Chi, web-based intervention, online, telehealth, unsupervised exercise, exercise, physical activity, arthritis, development, web based, hip, knee, gerontology, geriatric, older adult, aging, bone, workout, digital health, eHealth, literature review, telemedicine

## Abstract

**Background:**

Osteoarthritis is a leading contributor to global disability. While evidence supports the effectiveness of Tai Chi in improving symptoms for people with hip/knee osteoarthritis, access to in-person Tai Chi classes may be difficult for many people. An unsupervised online Tai Chi intervention for people with osteoarthritis can help overcome accessibility barriers. The Approach to Human-Centered, Evidence-Driven Adaptive Design (AHEAD) framework provides a practical guide for co-designing such an intervention.

**Objective:**

This study aims to develop an unsupervised online Tai Chi program for people with hip/knee osteoarthritis.

**Methods:**

An iterative process was conducted using the AHEAD framework. Initially, a panel of Tai Chi instructors and people with osteoarthritis was assembled. A literature review was conducted to inform the content of a survey (survey 1), which was completed by the panel and additional Australian Tai Chi instructors to identify Tai Chi movements for potential inclusion. Selection of Tai Chi movements was based on 3 criteria: those that were appropriate (for people with hip/knee osteoarthritis aged 45+ years), safe (to be performed at home unsupervised), and practical (to be delivered online using prerecorded videos). Movements that met these criteria were then ranked in a second survey (survey 2; using conjoint analysis methodology). Survey findings were discussed in a focus group, and the Tai Chi movements for program use were identified. A draft of the online Tai Chi program was developed, and a final survey (survey 3) was conducted with the panel to rate the appropriateness and safety of the proposed program. The final program was developed, and usability testing (think-aloud protocol) was conducted with people with knee osteoarthritis.

**Results:**

The panel consisted of 10 Tai Chi instructors and 3 people with osteoarthritis. The literature review identified Yang Style 24 as a common and effective Tai Chi style used in hip/knee osteoarthritis studies. Surveys 1 (n=35) and 2 (n=27) produced a ranked list of 24 Tai Chi movements for potential inclusion. This list was refined and informed by a focus group, with 10 Tai Chi movements being selected for inclusion (known as the Yang Style 10 form). Survey 3 (n=13) found that 92% (n=12) of the panel members believed that the proposed draft Tai Chi program was appropriate and safe, resulting in its adoption. The final program was produced and hosted on a customized website, “My Joint Tai Chi,” which was further refined based on user feedback (n=5). “My Joint Tai Chi” is currently being evaluated in a randomized controlled trial.

**Conclusions:**

This study demonstrates the use of the AHEAD framework to develop an unsupervised online Tai Chi intervention (“My Joint Tai Chi”) for people with hip/knee osteoarthritis. This intervention is now being tested for effectiveness and safety in a randomized controlled trial.

## Introduction

Osteoarthritis is a leading contributor to disability and chronic pain, with a global prevalence of 365 million in 2019 [1]. The knee is the most common lower limb site for osteoarthritis, followed by the hip [2]. Globally, it is estimated that cases of osteoarthritis will increase by 75% for the knee and nearly 80% for the hip by 2050 compared with 2020 [2]. People with hip and knee osteoarthritis often experience pain and impaired function, as well as comorbidities such as depression and anxiety [3,4].

All clinical guidelines for osteoarthritis recommend education and physical activity, including structured exercise, as the fundamental approach to treatment [5-7]. Tai Chi is specifically recommended as an exercise option for people with hip and knee osteoarthritis [5,7]. Tai Chi is a traditional Chinese, mind-body, land-based exercise that combines meditation with slow, gentle movements; deep diaphragmatic breathing; and relaxation [8]. Clinical trials indicate that Tai Chi is effective in improving pain, function, and quality of life for people with osteoarthritis [5,9-13]. With its meditative and mindfulness component, Tai Chi has also been shown to have broader benefits, such as improved psychological health, cognitive function, and sleep quality [14,15]. Tai Chi is generally performed in person in a group setting with demonstration and supervision from an instructor. However, this format can be difficult or inconvenient for many people to access, especially in regional or rural areas where there is a higher prevalence of people with osteoarthritis but Tai Chi classes may not be readily available [16].

An unsupervised online Tai Chi program may be a scalable way to help patients participate in Tai Chi in their own homes. Online exercise interventions have the potential to reach a wide audience regardless of location and at less cost to users and the health care system [17]. Since the COVID-19 pandemic, a number of Tai Chi schools and organizations now offer Tai Chi classes online [18-22]. These classes are generally conducted in a “synchronous” videoconferencing format with an instructor and require payment [23-26]. However, most Tai Chi programs are not specifically designed for people with osteoarthritis, which can make them challenging to perform correctly and safely [27]. Currently, there is no Tai Chi exercise program that is online, unsupervised, free, and tailored for people with lower limb osteoarthritis.

When designing interventions, the use of a theoretical framework is recommended to provide researchers with systematic and clear guidance [28,29]. The Approach to Human-Centered, Evidence-Driven Adaptive Design (AHEAD) is a 7-step framework that provides a practical guide for co-designing pragmatic and impactful health care interventions [30]. Using the AHEAD framework, this study aimed to develop an online, unsupervised Tai Chi program for people with hip and knee osteoarthritis.

## Methods

Development of the online Tai Chi program followed the 7 steps of the AHEAD framework, which are (1) define the problem and assemble a team, (2) review evidence, (3) seek inspiration, (4) synthesize, (5) develop guiding principles, (6) ideate, and (7) evaluate [30].

### Step A: Define the Problem and Assemble the Team

We defined the problem as the poor accessibility of evidence-based Tai Chi classes for people with osteoarthritis. To address this issue, we first assembled an advisory panel that included Tai Chi instructors and people with osteoarthritis. Tai Chi instructors were eligible to be part of the panel if they (1) were a member of a Tai Chi association in Australia and (2) had at least 5 years of experience teaching Tai Chi for adults (aged 45+ years) with musculoskeletal conditions. Potentially eligible instructors were identified by searching websites of Tai Chi associations. People with osteoarthritis were eligible if they had (1) self-reported symptomatic knee and/or hip osteoarthritis and (2) had participated in supervised Tai Chi exercise in the past year in Australia. It is suggested that around 10 people is an ideal size for a panel group discussion [31]. Therefore, we planned to recruit around 10 panel participants. Recruitment was conducted through email invitations to the Tai Chi instructors and email advertisements to our center’s consumer network. Interested instructors and people with osteoarthritis were asked to email the researchers and were followed up by phone to assess eligibility. A Plain Language Statement was provided to eligible panelists, and consent was obtained digitally through REDCap (Research Electronic Data Capture; Vanderbilt University). Gift vouchers of Aus $350 (US $235) were provided as an honorarium. To gain broader insights from the Tai Chi community, additional Tai Chi instructors were recruited Australia-wide to participate as nonpanel participants. Recruitment was done through the dissemination of study information by Presidents of Australian Tai Chi organizations, Facebook advertisements, and snowball sampling. Eligibility and consent procedures were consistent with panelist recruitment but with no remuneration provided.

### Step B: Gather Information—Review Evidence

There are many different styles of Tai Chi, each with different forms (a series of connected movements executed in a certain order) [32]. To identify the style and movements of Tai Chi to be considered for the online program, 1 researcher (SZ) first conducted a literature search (by Ovid, PubMed, and Google) to identify systematic reviews and randomized controlled trials (RCTs) evaluating the effects of Tai Chi in people with osteoarthritis and of online Tai Chi programs in any condition. The research team (RKN, KLB, RSH, and JH) discussed the findings from the literature search through meetings, phone calls, and emails and proposed a draft list of Tai Chi movements for potential inclusion in the unsupervised online Tai Chi program.

### Step C: Gather Information—Seek Inspiration

It was proposed by the Tai Chi instructor in the research team (JH) that the ideal number of Tai Chi movements to be included in an online program should be between 8 and 12, as it was thought more than 12 movements would be challenging to effectively learn and practice independently. To select the specific movements from those identified in Step B, 2 online surveys were conducted, involving both panel and nonpanel participants.

#### Survey 1

The purpose of this survey (conducted using REDCap software) was to identify which Tai Chi movements would be appropriate, safe, and practical for inclusion in the program. The survey asked participants to rate each identified Tai Chi movement (from Step B) against two criteria, which are (1) appropriateness of the movement for people with knee and/or hip osteoarthritis who are 45 years old and older when performed correctly, and (2) safety of the movement if performed at home unsupervised. Each movement was scored using separate 11-point numerical rating scales from 0 (not appropriate or safe at all) to 10 (completely appropriate or safe). Participants were also asked whether each Tai Chi movement would be practical to be delivered online using prerecorded videos that someone would watch at home (response options yes or no). Participants also provided feedback regarding Tai Chi exercise prescription, including the recommended length of each Tai Chi video session (response options 20, 30, 40, 50, and 60 minutes), weekly practice frequency (d/wk, range 1-7), and total weekly practice time (min/wk). Open-ended questions gathered additional suggestions for developing the program. The research team determined a priori that a Tai Chi movement would be retained for potential inclusion in the program if at least 70% of participants scored it at least 5 out of 10 for both appropriateness and safety, and it was deemed practical.

#### Survey 2

The purpose of this survey was to rank the Tai Chi movements retained from survey 1 so that 12 movements could be selected. Using a pairwise ranking technique (1000Minds) [33], participants were shown pairs of Tai Chi movements (selected based on those considered appropriate, safe, and practical from survey 1) and asked to identify which of the pair should take priority for inclusion in the program. The number of pairwise rankings required from each participant varied depending on their responses to the presented alternatives. The pairwise ranking process continued until the background mathematics established a ranked list of all Tai Chi movements for each participant and averaged over the sample.

### Step D: Synthesize

To synthesize information gathered from the first 2 surveys, an online focus group was conducted with panel participants, facilitated by the researchers (KLB, SZ, RKN, and JH).

Discussion topics included results of the first 2 surveys, practicalities of recording the online Tai Chi program, Tai Chi exercise prescription, and Tai Chi education content that would supplement the Tai Chi exercise program. The focus group discussion was recorded (both audio and visual) and transcribed verbatim. Notes and recordings were reviewed, replayed, and analyzed qualitatively to gather the panelist’s perspectives and to identify areas of consensus for the design of the Tai Chi program.

### Step E: Intervention Design—Develop and Apply Guiding Principles

The criteria of appropriateness, safety, and practicality were deemed as the guiding principles for this 12-week, online, unsupervised Tai Chi program. Based on the results from the first 2 surveys and focus group, a proposed 12-week Tai Chi program was filmed by the research team Tai Chi instructor (JH). Panelists were then sent the prototype video, through a final REDCap survey (survey 3). In this survey, they were asked to rate their level of agreement that the proposed program as a whole was (1) appropriate (for people aged 45+ years old with knee and/or hip osteoarthritis) and (2) safe (to be performed at home unsupervised). Each criterion was scored using a 5-point Likert scale from 1 (strongly disagree) to 5 (strongly agree). For the proposed program to be approved, 70% or more of participants were required to “strongly agree” or “agree” that the program was “appropriate” and “safe.” Since all movements included in the program had already been judged to be practical, it was unnecessary to vote on this.

### Step F: Intervention Design—Ideate (Brainstorm–Prototype–Test)

Finally, the 12-week, unsupervised, online Tai Chi program was filmed in a studio at the University of Melbourne. The produced videos were incorporated into a website prototype, “My Joint Tai Chi,” which was constructed by the research team and 2 research assistants. The website was based on our other evidence-based unsupervised exercise programs for knee and/or hip osteoarthritis “My Knee Exercise” [34] and “My Hip Exercise” [35] and developed in accordance with recommendations outlined by the Health on the Net Foundation’s Code of Conduct [36].

The website prototype underwent extensive usability testing with people with osteoarthritis, following a think-aloud protocol [37]. Volunteers from our previous osteoarthritis studies in Australia, who agreed to be contacted again, were invited through email to participate (by researcher SZ). Participants were eligible if they (1) had symptomatic knee osteoarthritis and (2) had no previous experience with Tai Chi exercise (in person or online) in the past 2 years. Purposive sampling was used to ensure diversity in age, sex, and symptom duration. A Plain Language Statement was provided, and consent was obtained digitally through REDCap. Participants received Aus $50 (US $35) gift vouchers as an honorarium.

Participants were scheduled for a 60-minute, one-on-one, online usability test with a researcher (SZ) through Zoom (Zoom Video Communications). During the usability test, participants were asked to share their screen and navigate the “My Joint Tai Chi” website prototype, as if they were commencing the Tai Chi program, while the researcher observed. Participants were encouraged to vocalize their thoughts on the website’s design, content, and functionality, with the researcher asking open-ended questions for further feedback. Notes on usability were taken and sessions were audio and video recorded. Field notes and recordings were qualitatively analyzed to identify usability issues, which were addressed before the next participant. This iterative process continued until no further issues were identified.

### Step G: Evaluate

The research team has designed and is currently conducting a 2-arm, parallel-design, superiority pragmatic RCT to assess the effectiveness of this unsupervised online Tai Chi intervention, (“My Joint Tai Chi”), compared with online osteoarthritis education control. Primary outcomes of self-reported pain during walking [38] and physical function [39] will be evaluated. A nested qualitative study has also been designed to explore the experience of people with osteoarthritis who use the “My Joint Tai Chi” intervention during the RCT. The RCT protocol and the results of the RCT will be published in separate subsequent papers.

### Ethical Considerations

The Human Research Ethics Committee at the University of Melbourne approved this study (2023-25788-36959-4). All data collected in this study was de-identified and stored on secure university servers, accessible only to the researchers using a password.

## Results

### Overview

A summary of the development of the “My Joint Tai Chi” intervention using the AHEAD Framework is provided in Table 1 [30].

**Table 1:** Summary of the design of the “My Joint Tai Chi” intervention using the AHEAD^a^ framework.

AHEAD framework domains	Intervention design stages
Define the problem and assemble the team (A)	Problem: Poor accessibility of evidence-based Tai Chi classes for people with osteoarthritis Team (n=17): Advisory panel including Tai Chi instructors (n=10) and people with osteoarthritis (n=3), and a research team including osteoarthritis researchers and/or physiotherapists (n=4; SZ, KLB, RKN, and RSH) and a Tai Chi instructor recruited from the panel.
Review evidence (B)	Literature review identified Yang Style 24 form as a common and effective form of Tai Chi for osteoarthritis. Twelve weeks was the most common length.
Seek inspiration (C)	Two surveys were completed by panel participants (Tai Chi instructors and people with osteoarthritis) and additional nonpanel Tai Chi instructors. Survey 1 (n=35) rated Yang Style 24 form and modifications (total 33 Tai Chi movements) based on appropriateness, safety, and practicality. A total of 24 movements identified as appropriate, safe, and practical. Survey 2 (n=27) produced a ranked list of the 24 movements retained from survey 1 (ranked based on most appropriate or safe to least appropriate or safe).
Synthesize (D)	Met with the panel (n=12) to discuss results from surveys and additional Tai Chi program design considerations. This resulted in the adoption of the Yang Style 10 form.
Develop and apply guiding principles (E)	Final survey with panel participants (n=13) rated a draft of the proposed program against the guiding principles of appropriateness (for people aged 45+ years with osteoarthritis) and safety (to be performed at home unsupervised). 92% (12/13) rated the program as both appropriate and safe.
Ideate (F)	A 12-week Tai Chi exercise program was developed, produced, and housed within a website prototype called “My Joint Tai Chi”. Usability testing was conducted with people with osteoarthritis (n=5) using a think-aloud protocol. Usability problems were identified, and the prototype was refined.
Evaluate (G)	The final “My Joint Tai Chi” intervention is currently being evaluated in a 2-arm, parallel-design, superiority pragmatic RCT^b^ (ACTRN12623000780651) and nested qualitative study. The methods and results of this RCT will be reported subsequently in separate papers.

^a^AHEAD: Approach to Human-Centered, Evidence-Driven Adaptive Design.

^b^RCT: randomized controlled trial.

### Step A: Define the Problem and Assemble the Team

We recruited 10 Tai Chi instructors and 3 people with osteoarthritis (1 with hip and knee osteoarthritis and 2 with knee osteoarthritis only) as advisory panel participants and another 22 Tai Chi instructors as nonpanel participants. One Tai Chi instructor (JH) was recruited to join the research team (that comprised osteoarthritis researchers and physiotherapists) to provide professional opinions about Tai Chi during the development process. Participant demographic information is provided in Table 2.

**Table 2:** Demographic characteristics of participants in Step A.

Participant characteristics				Survey 1 participants (n=35)		
				Panel (n=13)		Nonpanel (n=22)
**Tai Chi instructors, n**				10		22
	**Sex, female,** **n (%)**			6 (60)		11 (50)
	**Predominant style of Tai Chi taught, n (%)^a^**					
		Yang	9 (90)		19 (86)	
		Sun	5 (50)		5 (23)	
		Chen	2 (20)		1 (5)	
		Wu	1 (10)		1 (5)	
		Hao	2 (20)		0 (0)	
	**Years of experience teaching Tai Chi, mean (SD)**			24 (11)		16 (10)
	**Number of Tai Chi classes taught per week, mean (SD)**			8 (6)		3 (2)
**People with osteoarthritis, n**				3		—^b^
	**Sex, female,** **n (%)**			2 (67)		—
	**Age (years), mean (SD)**			73 (2)		—
	**Years of symptom duration, mean (SD)**			7 (3)		—
	**Years of Tai Chi practice, mean (SD)**			7 (2)		—

^a^Some Tai Chi instructors taught more than 1 style of Tai Chi.

^b^Not applicable.

### Step B: Gather Information—Review Evidence

Six systematic reviews and 14 RCTs were identified evaluating Tai Chi programs in people with osteoarthritis. Programs varied in length from 8 to 20 weeks, with 12 weeks being the most common [40-51]. The average Tai Chi exercise prescription was 3 Tai Chi sessions per week, each lasting 60 minutes [40-51]. Yang Style 24 form was the most commonly practiced [52]. This style was created in China in the 20th century and is known for its slow and graceful movements, with an emphasis on weight shifting in a wide stance [53]. It has been found to be effective in improving pain and physical function in people with osteoarthritis [54]. Hence, Yang Style was chosen as the foundational basis for the online Tai Chi program. There are 24 Tai Chi movements in the Yang Style 24 form. However, the Tai Chi instructor in the research team (JH) perceived some movements to be unsuitable for people with osteoarthritis and challenging to learn through online delivery, for example, movements that involve single-leg stance, maintained end-of-range hip and knee flexion, and multiple 180-degree turns. Therefore, the research team introduced 7 modifications and broke down certain movements, resulting in a total of 33 movements (Multimedia Appendix 1) for possible inclusion in the online program.

Four studies (2 feasibility trials, 1 longitudinal pilot study, and 1 RCT protocol) used telehealth-delivered Tai Chi for other chronic conditions such as long-term mobility disability, Parkinson disease, mild cognitive impairment, and cancer therapy–induced joint pain [23-25]. Programs were most commonly delivered synchronously using videoconferencing software Zoom (n=3). One study integrated Tai Chi training videos into a mobile phone app for people with Parkinson disease, which was connected to a clinician app for monitoring adherence [55]. Professional oversight through phone calls was also provided [55]. The Tai Chi for Arthritis program, endorsed by the US Centers for Disease Control and Prevention, does offer an online asynchronous course [56], but this has not been formally evaluated.

The research team determined that prerecorded videos of Tai Chi exercises would be the most convenient approach, allowing broader access without class scheduling or professional oversight. Moreover, it was decided to deliver the prerecorded Tai Chi videos by a dedicated website that also included osteoarthritis and Tai Chi education and exercise adherence support resources to allow for a potentially more effective multicomponent, osteoarthritis, digital, self-management intervention [57].

### Step C: Gather Information—Seek Inspiration

#### Survey 1

All 35 participants (13 panel and 22 nonpanel) completed survey 1 (Multimedia Appendix 1). Out of the 33 movements, 24 (73%) achieved consensus (defined as at least 70% of participants rating it at least 5 out of 10 for appropriateness, safety, and considering it to be practical). For the 9 excluded movements, refer to Multimedia Appendix 2. Additional Tai Chi exercise prescription suggestions are summarized in Table 3.

**Table 3:** Tai Chi exercise prescription suggestions from survey 1 (N=35).

Tai Chi exercise prescription suggestions		Value
**Length of each prerecorded video (min), n (%)**		
	30	17 (55)
	20	9 (29)
	40	2 (6)
	50	2 (6)
	60	1 (3)
**Frequency of Tai Chi practice (d/wk), n (%)**		
	3	12 (40)
	5	6 (20)
	4	4 (13)
	7	4 (13)
	2	3 (10)
	1	1 (3)
	6	0 (0)
**Weekly dosage of Tai Chi practice (total min/wk), mean (SD)**		114 (60)

#### Survey 2

As 24 movements met the criteria in survey 1 (exceeding the 12 required), a 1000Minds survey was conducted for further prioritization. A total of 27 participants (13 panel and 14 nonpanel) completed survey 2, which produced a ranked list of movements (Multimedia Appendix 3) for potential inclusion. Kendall W was 0.124, indicating high variance and low agreement among participants [58]. Thus, this ranked list was used only as a guide to inform further discussions with the panel participants by a focus group.

### Step D: Synthesize

Panel participants (12/13) were then involved in a 2-hour online focus group via Zoom. Suggestions provided during the focus group are listed in Multimedia Appendix 4. In summary, Tai Chi instructors advocated for the use of a recognized Tai Chi sequence, as opposed to solely relying on the ranked list of movements from the second survey (1000Minds). This is because the transition, stance, and flow between movements are vital in Tai Chi, rather than just presenting isolated “movements.” It was decided to use the modified Yang Style 10 form (Textbox 1) for the program because 7 out of the 10 movements aligned with the top 10 ranked movements in survey 2 and it is a recognized Tai Chi sequence [54]. The other 3 moves were ranked 13th, 16th, and 20th out of the 24 moves in the results of survey 2 (Multimedia Appendix 3). Modifications were also discussed for certain Tai Chi movements to ensure suitability for people with osteoarthritis and little previous Tai Chi experience. It was also decided that each 12-week video should include 5-10 minutes of Qigong exercise for warm-up and cooldown (an ancient wellness practice that is performed with minimal footwork [59]). All panelists agreed on the program duration (12 weeks), and it was decided that the program should start with 30- to 40-minute sessions and progress to 40- to 45-minute sessions to build endurance. Incorporating explanations of the martial applications of each selected Tai Chi movement and the practicalities of recording the program were discussed. Finally, resources for those wanting to continue Tai Chi practice after completing the online program were also suggested.

**The Yang Style 10 form Tai Chi movements.**

**Movement name**

- Commencement
- Repulse monkey
- Brush knee
- Part the wild horse’s mane
- Cloud hands
- Golden rooster stands on one leg
- Cross hands and kick
- Stroke peacock’s tail
- Embrace the tiger
- Closing

### Step E: Intervention Design—Develop and Apply Guiding Principles

All panel participants (n=13) took part in the final survey that involved voting on the draft program’s alignment with the guiding principles. A total of 92% (12/13) of participants rated “strongly agree” or “agree” to the draft 12-week Tai Chi program being (1) appropriate for people with knee and/or hip osteoarthritis and (2) safe to be performed at home unsupervised. The agreement reached the predetermined threshold (70%), and therefore, the draft online Tai Chi program was adopted as the final version.

### Step F: Intervention Design—Ideate (Brainstorm–Prototype–Test)

#### “My Joint Tai Chi” Website

**Figure 1:**
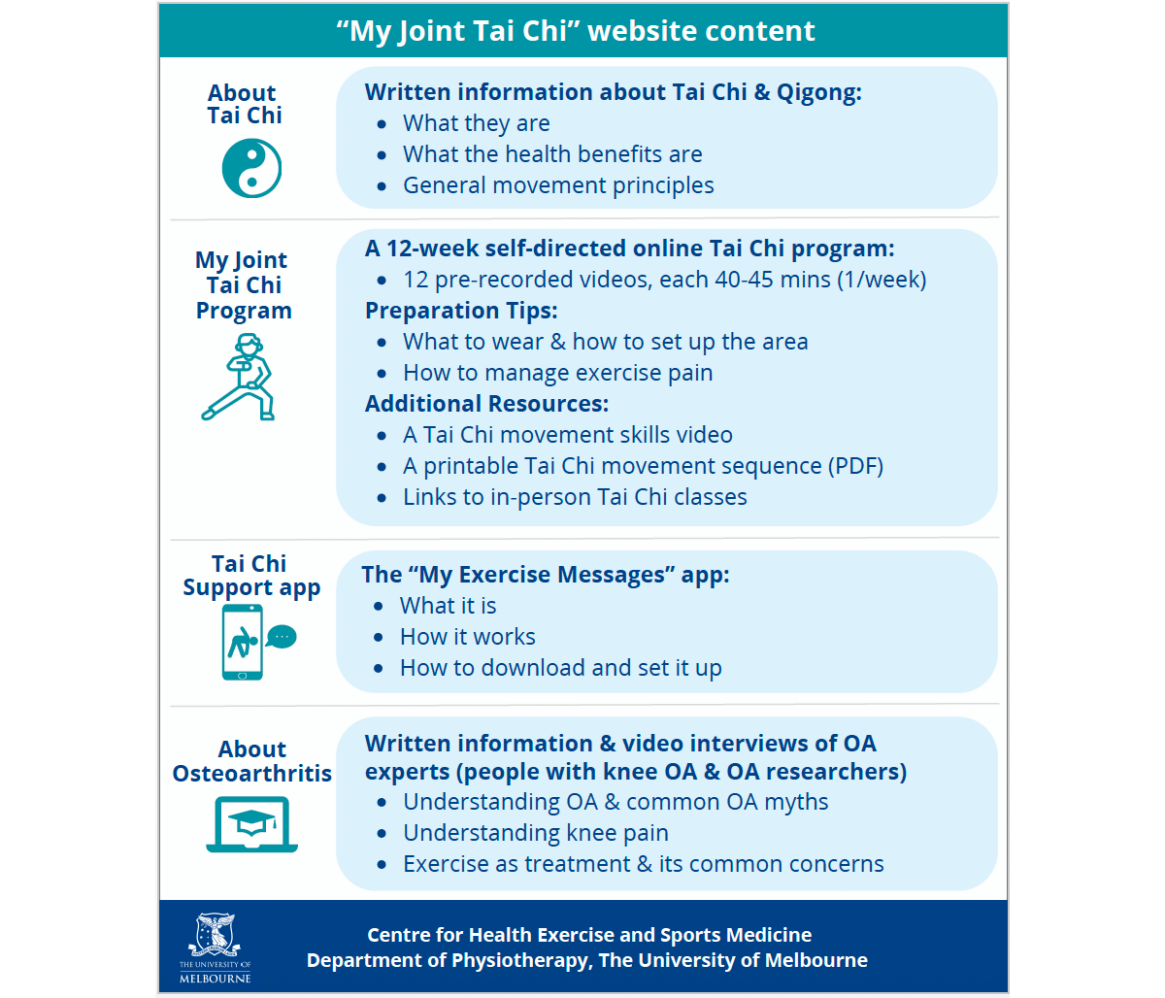
Description of the content in the 4 discrete sections of the “My Joint Tai Chi” website. OA: osteoarthritis.

The 12 professionally filmed videos showing a Tai Chi instructor (JH) demonstrating the Tai Chi movements were housed on a website called “My Joint Tai Chi” (the website was built specifically for research purposes and was not yet widely publicly available). “My Joint Tai Chi” contains a home page with a video tutorial explaining how to use the website and four sections including (1) the Tai Chi program, (2) information about Tai Chi, (3) information about osteoarthritis, and (4) instructions on how to access an exercise adherence app to support engagement with the Tai Chi program. Figure 1 outlines the contents of the website, and further detail is provided in Multimedia Appendix 5. Still images of modified Yang Style 10 form in the “My Joint Tai Chi” program are provided in Multimedia Appendix 6.

#### Usability Test

Five participants with osteoarthritis conducted website prototype usability testing. On average, they were 60 (SD 10) years old and had experienced knee pain for 2 (SD 1) years. Identified issues and implemented solutions are outlined in Multimedia Appendix 7. In summary, usability testing resulted in the redesign of the user interface and several pages for easier navigation. Page instructions were reworded for clearer and more explicit guidance.

### Step G: Evaluate

The RCT evaluating the “My Joint Tai Chi” intervention is currently recruiting and expected to complete data collection in February 2025. The RCT has been prospectively registered (ACTRN12623000780651). The nested qualitative study is anticipated to commence data collection in July 2024. The RCT protocol will be published separately.

## Discussion

### Principal Findings

This study reports the systematic codevelopment of an unsupervised online Tai Chi intervention for people with osteoarthritis using the AHEAD framework. The intervention is a 12-week, web-based Tai Chi program that is complemented with educational information about osteoarthritis and Tai Chi and supported by an app to facilitate Tai Chi exercise adherence. Once the RCT evaluation is complete (and subsequently any required modifications are made), the finalized program will be released online for public access at no cost to the user. The program has the potential to boost participation in physical activity among people with osteoarthritis.

This study has several strengths. First, the use of the AHEAD framework provided a structured foundation for the transparent and thorough reporting of intervention design and intervention components. This approach addresses the previous lack of clarity in intervention development, with evidence showing that most Tai Chi studies do not meet the expected intervention reporting standards [60-62]. Second, by using an evidence-based, iterative, and robust process, we were able to incorporate opinions from a broad panel of Tai Chi experts and people with osteoarthritis (n=35 in total), along with physiotherapists and osteoarthritis researchers. To the best of our knowledge, only one other study has incorporated end-user feedback (n=14) into the design of an online, seated Tai Chi program for people with mobility disabilities [25]. However, that program was designed to include remote supervision, and its effectiveness has not been tested in an RCT [25]. Hence, no previous unsupervised online Tai Chi program has been codeveloped by such a large sample group nor subjected to rigorous evaluation. Third, since this Tai Chi exercise program was designed for people with knee and/or hip osteoarthritis, the panel also deemed it appropriate for people 45 years of age and older who do not have osteoarthritis. Hence, the Tai Chi exercise program videos developed could potentially be used as a strategy to increase access to Tai Chi exercise for a broader audience.

One limitation is that there were only 3 people with osteoarthritis out of a total of 35 participants involved in Step C (Seek Inspiration), indicating a potential underrepresentation of people with osteoarthritis. However, these 3 people provided constructive feedback in Step D (Synthesize), and an additional 5 people with osteoarthritis were involved in the final website usability testing in Step F. Another potential limitation is that we have developed an intervention that requires access to digital technologies and technological literacy. To fully gain the benefit from the “My Joint Tai Chi” program, the user is required to have a computer with internet access and preferably to have a mobile phone (to use the exercise support app). However, given that 94% of Australian households have a computer with 86% of them having internet access [63], and that 78% of Australian adults 65 years of age and older use a mobile phone [64], this suggests that the use of “My Joint Tai Chi” website is broadly accessible by most Australians.

### Conclusions

A systematic design approach using the AHEAD framework was successful in developing this user-centered intervention and may serve as a guide for others developing unsupervised digital interventions. To our knowledge, “My Joint Tai Chi” is the first unsupervised online Tai Chi intervention designed for people with osteoarthritis. The program is now being evaluated in an RCT that will provide insights into the effectiveness and safety of the program compared with online osteoarthritis education control. The prerecorded Tai Chi exercise videos in the program can also be used to increase physical activity for people without osteoarthritis in the community at large.

## Appendix

Multimedia Appendix 1 Survey 1, including the 33 Tai Chi movements for potential inclusion in the Tai Chi program.

Multimedia Appendix 2 The 9 movements excluded from survey 1.

Multimedia Appendix 3 The ranked list of Tai Chi movements based on survey 2.

Multimedia Appendix 4 Suggestions and recommendation from focus group discussion (n=12).

Multimedia Appendix 5 “My Joint Tai Chi” website section description.

Multimedia Appendix 6 Still images of modified Yang Style 10 form in the final “My Joint Tai Chi” program.

Multimedia Appendix 7 Usability issues identified and corresponding implemented solutions.

## Abbreviations

AHEAD: Approach to Human-Centered, Evidence-Driven Adaptive Design
RCT: randomized controlled trial
REDCap: Research Electronic Data Capture
