# Supplementary material for: Development of a 12-Week Unsupervised Online Tai Chi Program for People With Hip and Knee Osteoarthritis: Mixed Methods Study
Source: JMIR Aging. 2024 Sep 30;7:e55322. doi: 10.2196/55322 (PMC11474117; doi:10.2196/55322)
Supplement: Multimedia Appendix 1 [file aging_v7i1e55322_app1.pdf]

My Joint Tai Chi Development Survey 1

Thank you [first_name_consent], for taking the time to work through the following questionnaire in preparation for our first panel meeting. The research team greatly appreciates your time and effort in ensuring that the program we develop and include in an upcoming clinical trial is as polished, appropriate, and effective as possible for our study participants and for people in our communities managing hip and knee osteoarthritis.

The following pages should take you approximately 30 minutes to work through and will request some basic information about yourself and your consideration and feedback on a variety of forms that have been used in traditional Tai Chi.

Please select which of the following best describes you

◯ Tai Chi instructor panelist ◯ Consumer panelist

How old are you? (please provide in numbers only e.g. 56) __________________________

What was your sex recorded at birth?
◯ Male
◯ Female
◯ Another term (please specify)

Another term (please specify) __________________________

How do you describe your gender?
◯ Man or male
◯ Woman or female
◯ Non-binary
◯ I use a different term (please specify)
◯ Prefer not to answer

I use a different term (please specify) __________________________

How many years of experience of teaching Tai Chi do you have? (please answer using a number) __________________________

How many TaiChi classes do you teach in an average week? (please answer using a number) __________________________

What are the predominant styles of Tai Chi you teach? (select all that apply)
☐ Yang
☐ Sun
☐ Chen
☐ Wu
☐ Hao
☐ Other

Other, please list what these are __________________________

Are you a member of any of the following professional Tai Chi organisations? (Please select all that apply to you from the following professional TaiChi Registration/ Accreditation):

- ☐ Kung Fu Wushu Australia (KWA): The administration of the National Coaching Accreditation Scheme (NCAS) for Kung Fu/Wu Shu. It is officially recognised by the Australian Sports Commission as the peak organisation governing the interests of Kung Fu/Wu Shu in Australia.
- ☐ TaiChi Association of Australia (TCAA): A member organisation of KWA
- ☐ Wushu Tai Chi & Qigong Australia (WTQA): An independent, inclusive, not for-profit association with the principal aim of promoting the benefits & improving the standards of these arts across Australia
- ☐ Tai Chi for Health Institute (TCHI): Founded by Dr Paul Lam with the purpose of empowering people to improve health and wellness through the Tai Chi for Health programs
- ☐ International Wushu Federation (IWUF): International sport organization and is the governing body for wushu in all its forms worldwide. It is recognized by the International Olympic Committee (IOC)
- ☐ Chinese Wushu Association
- ☐ Others

Others: (please list) ______________________________

What is your most painful joint? Select one option:

○ Right knee ○ Left knee ○ Right hip ○ Left hip

How long have you been experiencing symptoms in your [painful_joint]? (provide response in years e.g. 5.5 years) ______________________________

How long have you been practicing Tai Chi? (provide response in years e.g. 2.5 years) ______________________________

Guiding Principles & Goals:

Our primary goal is to develop a 12-week evidence-based unsupervised online Tai Chi program that is:

appropriate for people with knee and/or hip osteoarthritis who are typically over the age of 45 and have painful joints with likely impaired function safe to be performed at home unsupervised, and practical to be delivered online at home using pre-recorded videos. For example, the Tai Chi program doesn't involve movement that requires people to turn their back to the computer screen for long periods of time, making it hard to watch the instructor/video, and/or the movement doesn't require too much room space The program aims to:

Reduce hip and knee pain Improve function and ability to perform daily activities Target Population:

When working through the movements for review, please consider the following with regards to our population:

People with hip and/or knee osteoarthritis who are typically   aged 45 years or over and have activity-related pain at the affected joint;   Likely have no prior experience of Tai Chi exercises or classes Tai Chi Program Outline:

The 12-week Tai Chi program will comprise a set of pre-recorded videos (1 video per week). The videos will be housed in a website (MyJoint Tai Chi) which will also contain educational information about osteoarthritis and about Tai Chi. The entire online program will be self-directed. There will be no contact with a Tai Chi instructor or clinician. There will be 12 videos (1 video per week, each 45-60 minutes) and it will be recommended that people perform the exercise in each weekly video at least 3 times for that week No exercise equipment will be required Instructions:

Research shows that Yang style 24 forms are the most commonly used forms in Tai Chi related studies. The following simplified 24 Tai Chi Movements are extracted from Simplified Tai Chi Chuan: 24 postures with applications and standard 48 postures by Shou-Yu Liang, Wen-Ching Wu. Modified movements are also provided.

We ask you to evaluate each movement using three criteria: Appropriateness, Safety, and Practicality.

Please consider these definitions:

Appropriateness: How appropriate is the movement for people with knee/hip osteoarthritis who are over 45 years old when done correctly. Appropriateness will be rated on a scale from 0 to 10 (0 = Not at all appropriate for people with hip/knee OA; 10=Completely appropriate for people with hip/knee OA when done correctly) - for this criterion, please select the one number on the scale that best represents your opinion. Safety: How safe is the movement if it were to be performed at home unsupervised when done correctly. Safety will be rated on a scale from 0 to 10 (0 = Not at all safe for people to perform at home unsupervised; 10=Completely safe for people to perform at home unsupervised when done correctly) - for this criterion, please select the one number on the scale that best represents your opinion. Practicality: Is the movement practical to be delivered online using pre-recorded videos that someone will watch at home. For example, the movement would not be practical if it required people to turn their back to the computer screen for long periods of time, making it hard to view the instructor/video. The movement would not be practical if it required too much room space. Practicality will be rated by selecting either Yes or No. Let's get started...

Section 1

Movement 1 Commencing Posture

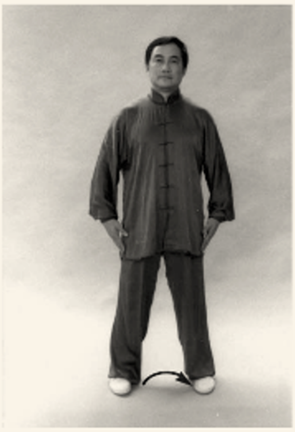

Bend your knees slightly. Then step to your left with your left leg, shoulder width apart. (Begin to inhale.)

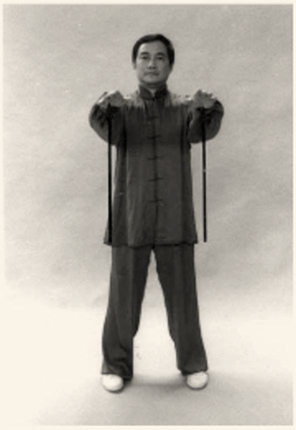

Rotate your palms as you raise your arms up slowly to shoulder level, palms facing down.

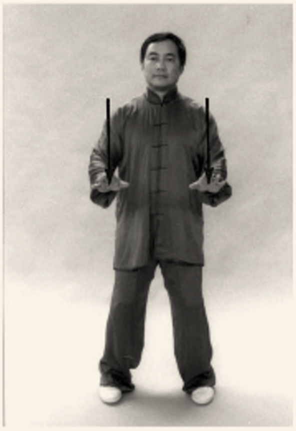

Pull your arms in slightly and lower them to abdomen level as you bend your knees slightly. (Exhale.)

Is this movement appropriate for people with knee/hip osteoarthritis when done correctly?
Please select the one number on the scale that best represents your opinion with 0 = "Not at all appropriate for people with hip/knee OA" and 10 = "Completely appropriate for people with hip/knee OA when done correctly"

- ◯ 0 = Not at all appropriate for people with hip/knee OA
- ◯ 1
- ◯ 2
- ◯ 3
- ◯ 4
- ◯ 5
- ◯ 6
- ◯ 7
- ◯ 8
- ◯ 9
- ◯ 10 = Completely appropriate for people with hip/knee OA when done correctly

---

Is this movement safe to do unsupervised at home when done correctly?
Please select the one number on the scale that best represents your opinion with 0 = "Not at all safe for people to perform at home unsupervised" and 10= "Completely safe for people to perform at home unsupervised when done correctly"

- ◯ 0 = Not at all safe for people to perform at home unsupervised
- ◯ 1
- ◯ 2
- ◯ 3
- ◯ 4
- ◯ 5
- ◯ 6
- ◯ 7
- ◯ 8
- ◯ 9
- ◯ 10 = Completely safe for people to perform at home unsupervised when done correctly

---

Is this movement practical to be delivered online using pre-recorded videos that someone will watch at home?

For example: the movement would not be practical if it involved movements that require people to turn their back to the computer screen for long periods of time, making it hard to view the instructor/video, and/or if the movements required too much room space

- ◯ Yes
- ◯ No

Section 2

Movement 2 The Wild Horse Parts Its Mane, left side, right side

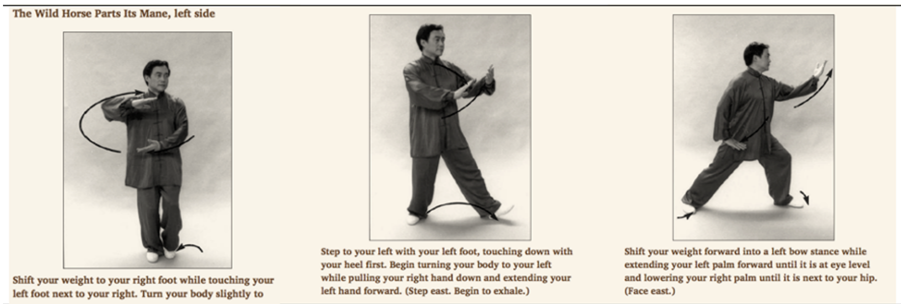

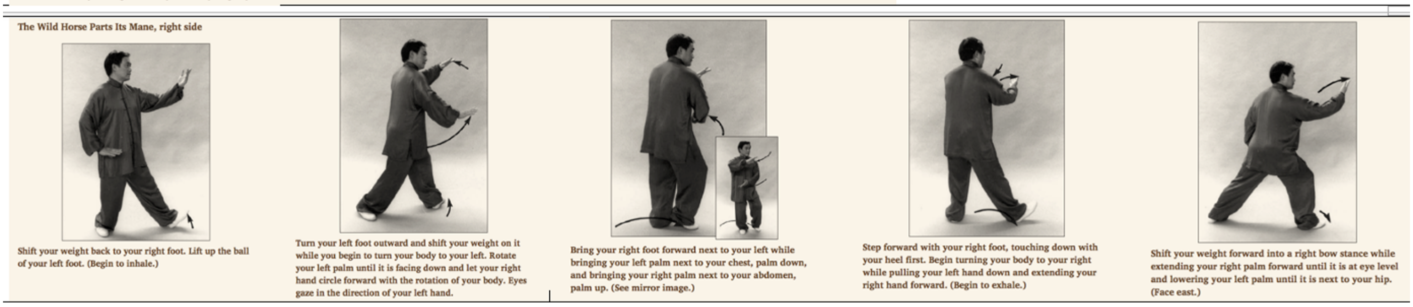

Is this movement appropriate for people with knee/hip osteoarthritis when done correctly?
Please select the one number on the scale that best represents your opinion with 0 = "Not at all appropriate for people with hip/knee OA" and 10 = "Completely appropriate for people with hip/knee OA when done correctly"

- ◯ 0 = Not at all appropriate for people with hip/knee OA
- ◯ 1
- ◯ 2
- ◯ 3
- ◯ 4
- ◯ 5
- ◯ 6
- ◯ 7
- ◯ 8
- ◯ 9
- ◯ 10 = Completely appropriate for people with hip/knee OA when done correctly

Is this movement safe to do unsupervised at home when done correctly?
Please select the one number on the scale that best represents your opinion with 0 = "Not at all safe for people to perform at home unsupervised" and 10= "Completely safe for people to perform at home unsupervised when done correctly"

- ◯ 0 = Not at all safe for people to perform at home unsupervised
- ◯ 1
- ◯ 2
- ◯ 3
- ◯ 4
- ◯ 5
- ◯ 6
- ◯ 7
- ◯ 8
- ◯ 9
- ◯ 10 = Completely safe for people to perform at home unsupervised when done correctly

Is this movement practical to be delivered online using pre-recorded videos that someone will watch at home?

For example: the movement would not be practical if it involved movements that require people to turn their back to the computer screen for long periods of time, making it hard to view the instructor/video, and/or if the movements required too much room space

- ◯ Yes
- ◯ No

Section 3

Movement 3 White Crane Spreads Its Wings

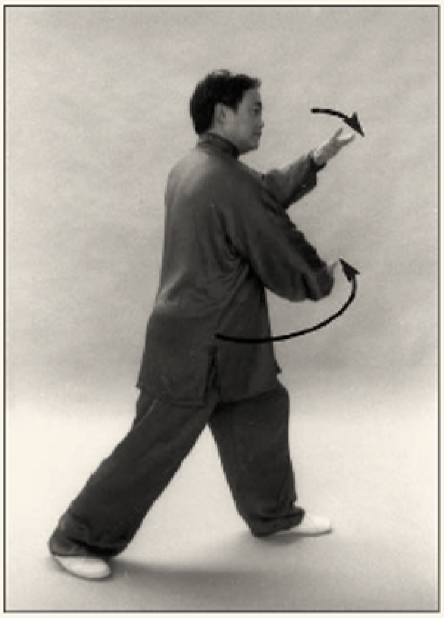

Turn your body slightly to your left while extending your right palm forward and rotating both palms until they face each other. Right palm faces up; left palm faces down. (Inhale.)

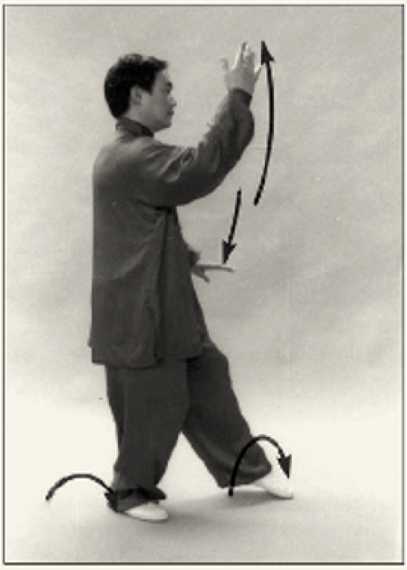

Bring your right foot a half step forward. Shift all your weight onto your right foot. At the same time, bring your right palm up past your left elbow and lift your left foot up slightly. Complete the movement by lowering your left palm down to waist level, palm down. Raise your right palm up to head level, palm facing inward, and

---

Is this movement appropriate for people with knee/hip osteoarthritis when done correctly?
Please select the one number on the scale that best represents your opinion with 0 = "Not at all appropriate for people with hip/knee OA" and 10 = "Completely appropriate for people with hip/knee OA when done correctly"

- ◯ 0 = Not at all appropriate for people with hip/knee OA
- ◯ 1
- ◯ 2
- ◯ 3
- ◯ 4
- ◯ 5
- ◯ 6
- ◯ 7
- ◯ 8
- ◯ 9
- ◯ 10 = Completely appropriate for people with hip/knee OA when done correctly

---

Is this movement safe to do unsupervised at home when done correctly?
Please select the one number on the scale that best represents your opinion with 0 = "Not at all safe for people to perform at home unsupervised" and 10= "Completely safe for people to perform at home unsupervised when done correctly"

- ◯ 0 = Not at all safe for people to perform at home unsupervised
- ◯ 1
- ◯ 2
- ◯ 3
- ◯ 4
- ◯ 5
- ◯ 6
- ◯ 7
- ◯ 8
- ◯ 9
- ◯ 10 = Completely safe for people to perform at home unsupervised when done correctly

Is this movement practical to be delivered online using pre-recorded videos that someone will watch at home?

For example: the movement would not be practical if it involved movements that require people to turn their back to the computer screen for long periods of time, making it hard to view the instructor/video, and/or if the movements required too much room space

◯ Yes
◯ No

Section 4

Movement 4 Brush knee and step forward, left side, ride side

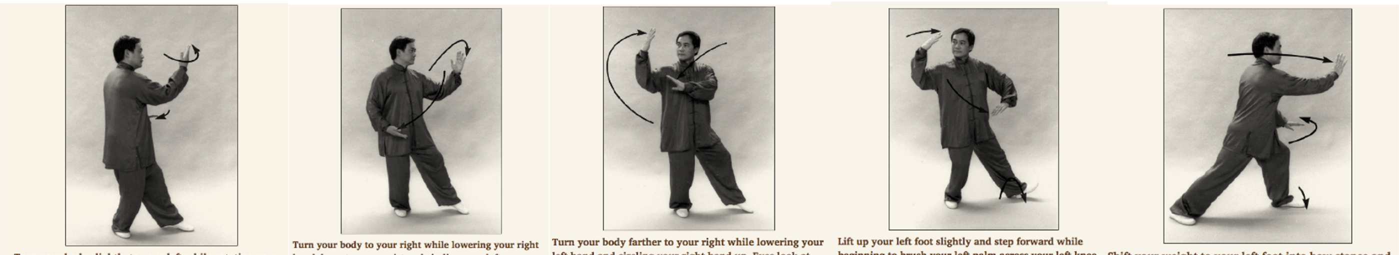

Turn your body slightly to your left while rotating your right palm to face you. (Inhale.)

Turn your body to your right while lowering your right hand down to your waist and circling your left arm up with your palm facing to your right. (Exhale.)

Turn your body farther to your right while lowering your left hand and circling your right hand up. Eyes look at your right palm. (Inhale.)

Lift up your left foot slightly and step forward while beginning to brush your left palm across your left knee, and extend your right palm forward. (Begin to exhale.)

Shift your weight to your left foot into bow stance and extend your right palm forward.

Brush Knee and Step Forward, right side

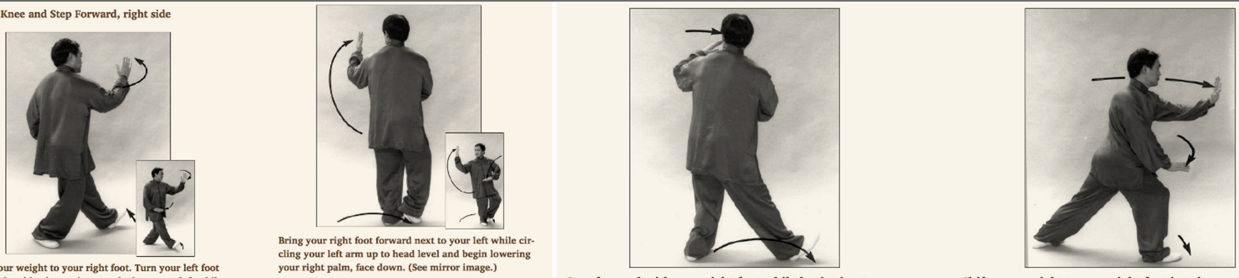

Shift your weight to your right foot. Turn your left foot outward and begin turning your body to your left while rotating your right palm inward and pulling your left palm next to your abdomen. (See mirror image. Begin to inhale.)

Bring your right foot forward next to your left while circling your left arm up to head level and begin lowering your right palm, face down. (See mirror image.)

Step forward with your right foot while beginning to brush your right palm across your right knee and extend your left palm forward. (Begin to exhale.)

Shift your weight to your right foot into bow stance and extend your left palm forward.

Is this movement appropriate for people with knee/hip osteoarthritis when done correctly?
Please select the one number on the scale that best represents your opinion with 0 = "Not at all appropriate for people with hip/knee OA" and 10 = "Completely appropriate for people with hip/knee OA when done correctly"

- ◯ 0 = Not at all appropriate for people with hip/knee OA
- ◯ 1
- ◯ 2
- ◯ 3
- ◯ 4
- ◯ 5
- ◯ 6
- ◯ 7
- ◯ 8
- ◯ 9
- ◯ 10 = Completely appropriate for people with hip/knee OA when done correctly

Is this movement safe to do unsupervised at home when done correctly?
Please select the one number on the scale that best represents your opinion with 0 = "Not at all safe for people to perform at home unsupervised" and 10= "Completely safe for people to perform at home unsupervised when done correctly"

- ◯ 0 = Not at all safe for people to perform at home unsupervised
- ◯ 1
- ◯ 2
- ◯ 3
- ◯ 4
- ◯ 5
- ◯ 6
- ◯ 7
- ◯ 8
- ◯ 9
- ◯ 10 = Completely safe for people to perform at home unsupervised when done correctly

Is this movement practical to be delivered online using pre-recorded videos that someone will watch at home?<br><br>For example: the movement would not be practical if it involved movements that require people to turn their back to the computer screen for long periods of time, making it hard to view the instructor/video, and/or if the movements required too much room space	◯ Yes<br>◯ No

Section 5

Movement 5 Playing the Lute

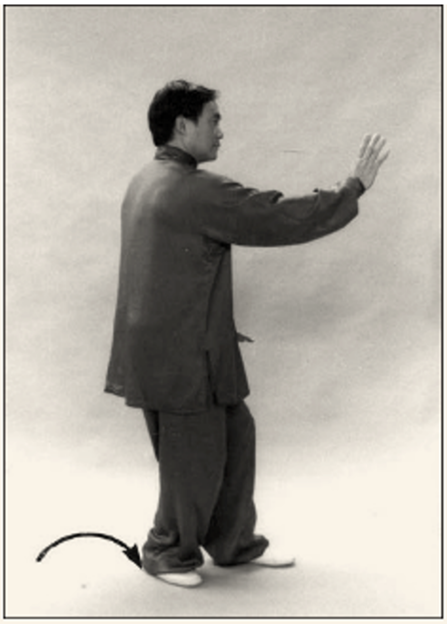

Bring your right foot a half step forward and shift your weight on it. (Inhale.)

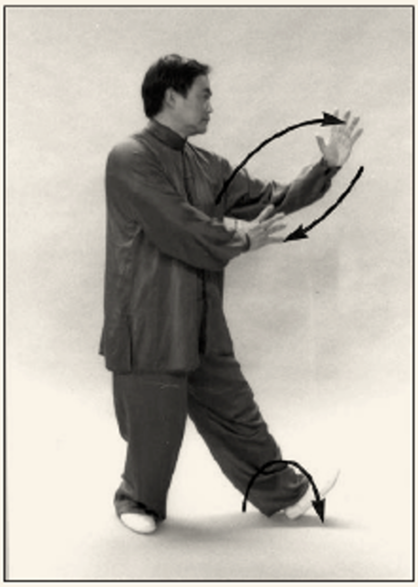

Lift up your left foot and touch down with your heel while circling your right palm down and circling your left palm forward. Both palms face inward. (Exhale.)

Is this movement appropriate for people with knee/hip osteoarthritis when done correctly?
Please select the one number on the scale that best represents your opinion with 0 = "Not at all appropriate for people with hip/knee OA" and 10 = "Completely appropriate for people with hip/knee OA when done correctly"

- ◯ 0 = Not at all appropriate for people with hip/knee OA
- ◯ 1
- ◯ 2
- ◯ 3
- ◯ 4
- ◯ 5
- ◯ 6
- ◯ 7
- ◯ 8
- ◯ 9
- ◯ 10 = Completely appropriate for people with hip/knee OA when done correctly

Is this movement safe to do unsupervised at home when done correctly?
Please select the one number on the scale that best represents your opinion with 0 = "Not at all safe for people to perform at home unsupervised" and 10= "Completely safe for people to perform at home unsupervised when done correctly"

- ◯ 0 = Not at all safe for people to perform at home unsupervised
- ◯ 1
- ◯ 2
- ◯ 3
- ◯ 4
- ◯ 5
- ◯ 6
- ◯ 7
- ◯ 8
- ◯ 9
- ◯ 10 = Completely safe for people to perform at home unsupervised when done correctly

Is this movement practical to be delivered online using pre-recorded videos that someone will watch at home?

For example: the movement would not be practical if it involved movements that require people to turn their back to the computer screen for long periods of time, making it hard to view the instructor/video, and/or if the movements required too much room space

◯ Yes
◯ No

Section 6

Movement 6 Repulse Monkey, right side, left side

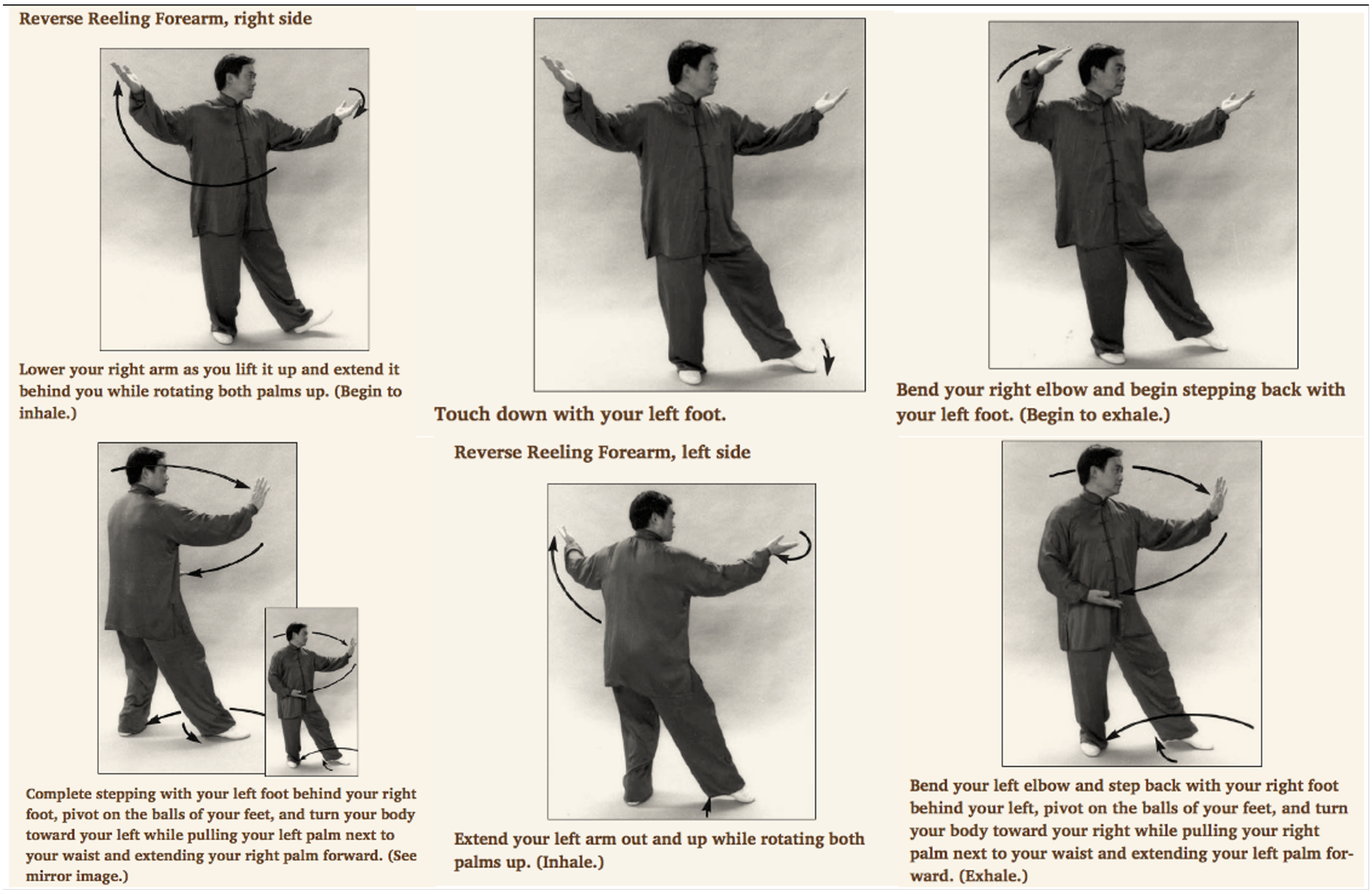

Is this movement appropriate for people with knee/hip osteoarthritis when done correctly?<br>Please select the one number on the scale that best represents your opinion with 0 = "Not at all appropriate for people with hip/knee OA" and 10 = "Completely appropriate for people with hip/knee OA when done correctly"	◯ 0 = Not at all appropriate for people with hip/knee OA<br>◯ 1<br>◯ 2<br>◯ 3<br>◯ 4<br>◯ 5<br>◯ 6<br>◯ 7<br>◯ 8<br>◯ 9<br>◯ 10 = Completely appropriate for people with hip/knee OA when done correctly
Is this movement safe to do unsupervised at home when done correctly?<br>Please select the one number on the scale that best represents your opinion with 0 = "Not at all safe for people to perform at home unsupervised" and 10= "Completely safe for people to perform at home unsupervised when done correctly"	◯ 0 = Not at all safe for people to perform at home unsupervised<br>◯ 1<br>◯ 2<br>◯ 3<br>◯ 4<br>◯ 5<br>◯ 6<br>◯ 7<br>◯ 8<br>◯ 9<br>◯ 10 = Completely safe for people to perform at home unsupervised when done correctly

Is this movement practical to be delivered online using pre-recorded videos that someone will watch at home?

For example: the movement would not be practical if it involved movements that require people to turn their back to the computer screen for long periods of time, making it hard to view the instructor/video, and/or if the movements required too much room space

◯ Yes
◯ No

Section 7

Movement 7 Grasp Sparrow's Tail Left side

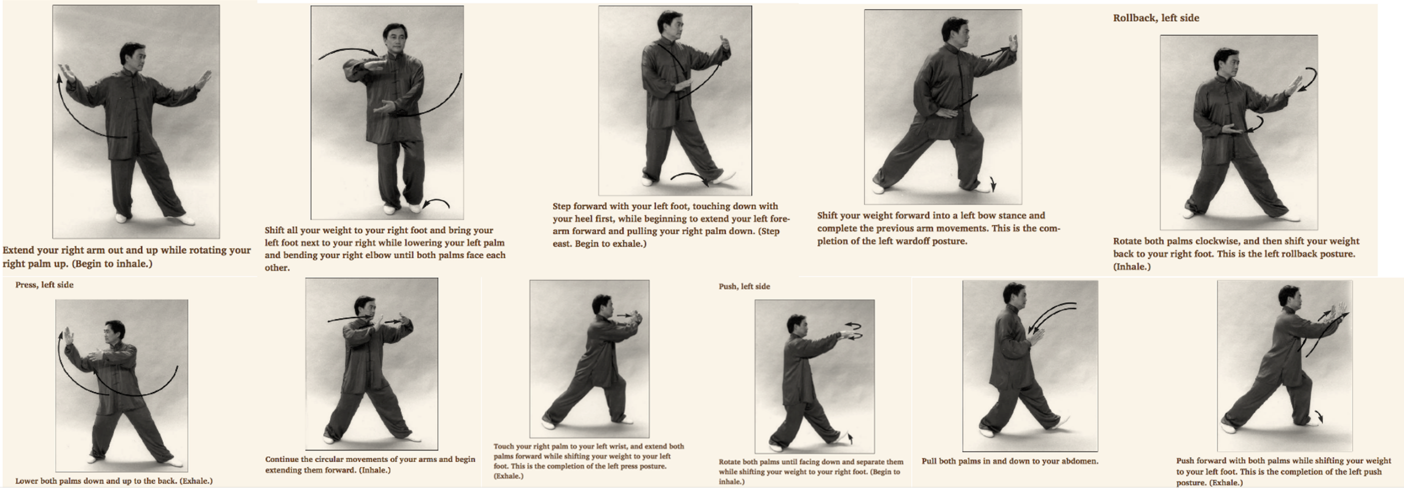

Is this movement appropriate for people with knee/hip osteoarthritis when done correctly?
Please select the one number on the scale that best represents your opinion with 0 = "Not at all appropriate for people with hip/knee OA" and 10 = "Completely appropriate for people with hip/knee OA when done correctly"

◯ 0 = Not at all appropriate for people with hip/knee OA
◯ 1
◯ 2
◯ 3
◯ 4
◯ 5
◯ 6
◯ 7
◯ 8
◯ 9
◯ 10 = Completely appropriate for people with hip/knee OA when done correctly

Is this movement safe to do unsupervised at home when done correctly?
Please select the one number on the scale that best represents your opinion with 0 = "Not at all safe for people to perform at home unsupervised" and 10= "Completely safe for people to perform at home unsupervised when done correctly"

◯ 0 = Not at all safe for people to perform at home unsupervised
◯ 1
◯ 2
◯ 3
◯ 4
◯ 5
◯ 6
◯ 7
◯ 8
◯ 9
◯ 10 = Completely safe for people to perform at home unsupervised when done correctly

Is this movement practical to be delivered online using pre-recorded videos that someone will watch at home?

For example: the movement would not be practical if it involved movements that require people to turn their back to the computer screen for long periods of time, making it hard to view the instructor/video, and/or if the movements required too much room space

◯ Yes
◯ No

Section 8

Movement 8 Grasp Sparrow's Tail Right side

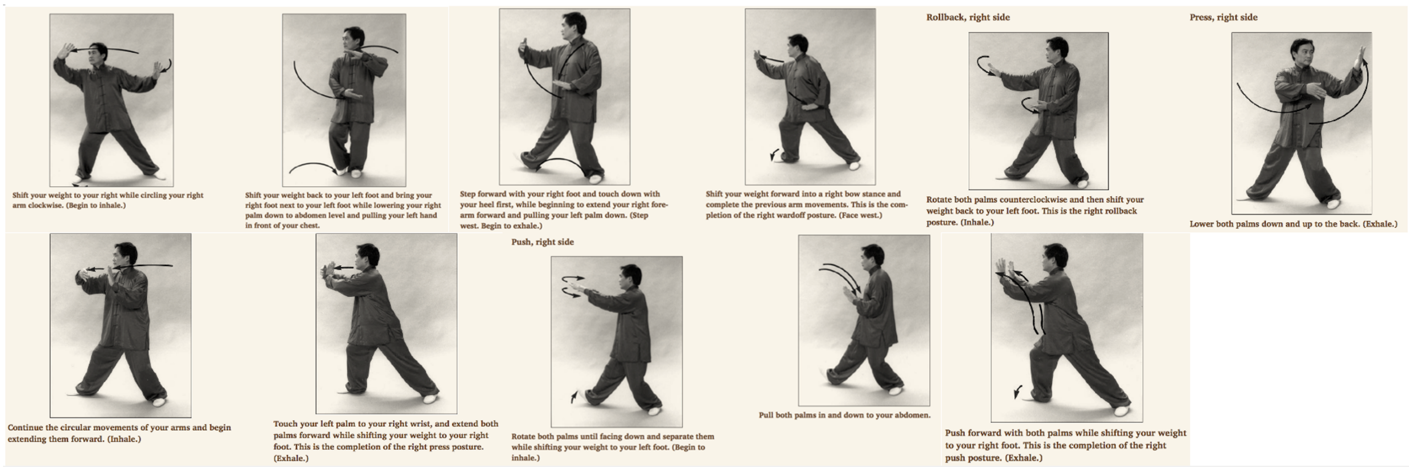

Is this movement appropriate for people with knee/hip osteoarthritis when done correctly?
Please select the one number on the scale that best represents your opinion with 0 = "Not at all appropriate for people with hip/knee OA" and 10 = "Completely appropriate for people with hip/knee OA when done correctly"

- ◯ 0 = Not at all appropriate for people with hip/knee OA
- ◯ 1
- ◯ 2
- ◯ 3
- ◯ 4
- ◯ 5
- ◯ 6
- ◯ 7
- ◯ 8
- ◯ 9
- ◯ 10 = Completely appropriate for people with hip/knee OA when done correctly

---

Is this movement safe to do unsupervised at home when done correctly?
Please select the one number on the scale that best represents your opinion with 0 = "Not at all safe for people to perform at home unsupervised" and 10= "Completely safe for people to perform at home unsupervised when done correctly"

- ◯ 0 = Not at all safe for people to perform at home unsupervised
- ◯ 1
- ◯ 2
- ◯ 3
- ◯ 4
- ◯ 5
- ◯ 6
- ◯ 7
- ◯ 8
- ◯ 9
- ◯ 10 = Completely safe for people to perform at home unsupervised when done correctly

---

Is this movement practical to be delivered online using pre-recorded videos that someone will watch at home?

For example: the movement would not be practical if it involved movements that require people to turn their back to the computer screen for long periods of time, making it hard to view the instructor/video, and/or if the movements required too much room space

- ◯ Yes
- ◯ No

Section 9

Movement 9 Single Whip

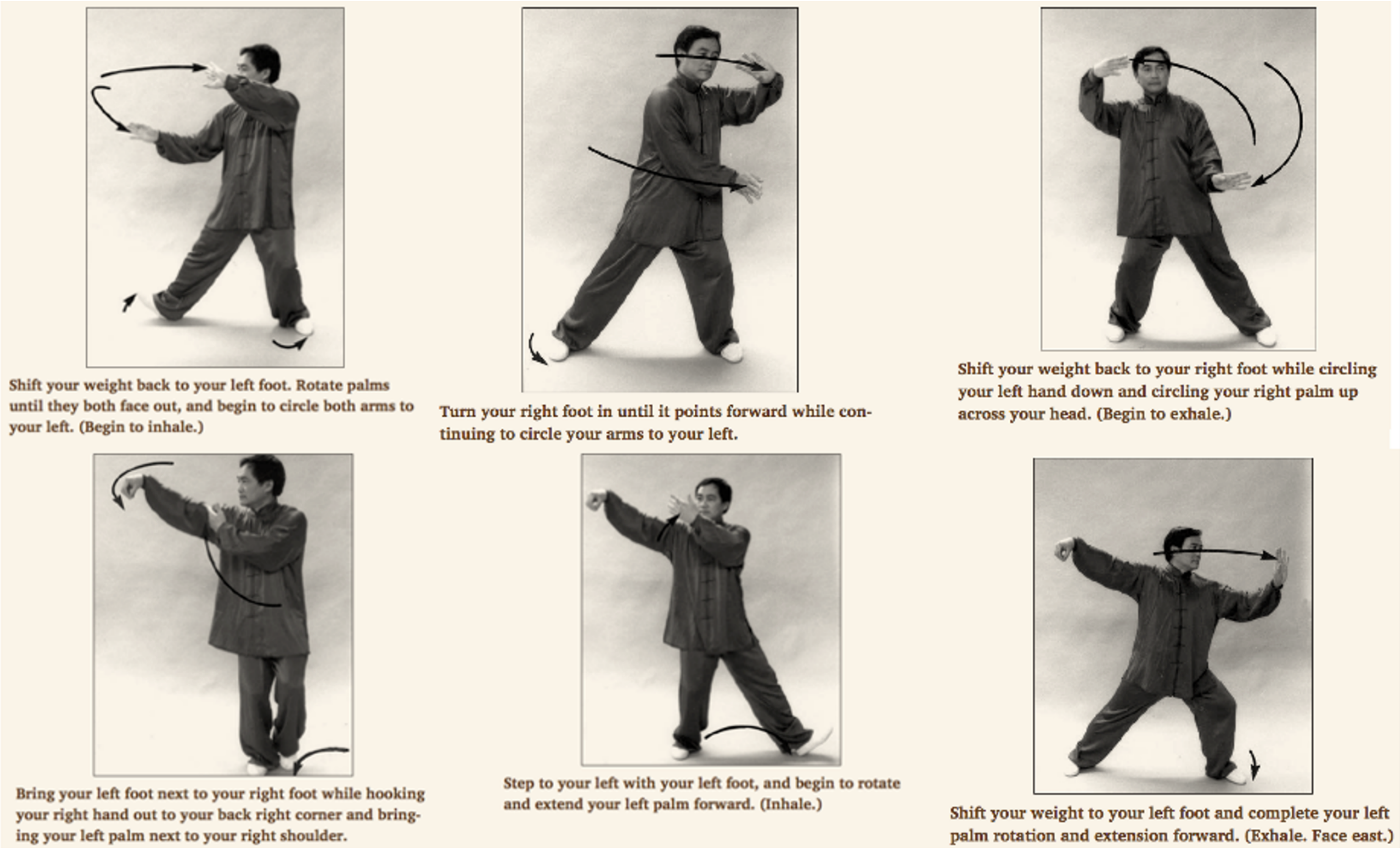

Is this movement appropriate for people with knee/hip osteoarthritis when done correctly?
Please select the one number on the scale that best represents your opinion with 0 = "Not at all appropriate for people with hip/knee OA" and 10 = "Completely appropriate for people with hip/knee OA when done correctly"

- ◯ 0 = Not at all appropriate for people with hip/knee OA
- ◯ 1
- ◯ 2
- ◯ 3
- ◯ 4
- ◯ 5
- ◯ 6
- ◯ 7
- ◯ 8
- ◯ 9
- ◯ 10 = Completely appropriate for people with hip/knee OA when done correctly

---

Is this movement safe to do unsupervised at home when done correctly?
Please select the one number on the scale that best represents your opinion with 0 = "Not at all safe for people to perform at home unsupervised" and 10= "Completely safe for people to perform at home unsupervised when done correctly"

- ◯ 0 = Not at all safe for people to perform at home unsupervised
- ◯ 1
- ◯ 2
- ◯ 3
- ◯ 4
- ◯ 5
- ◯ 6
- ◯ 7
- ◯ 8
- ◯ 9
- ◯ 10 = Completely safe for people to perform at home unsupervised when done correctly

Is this movement practical to be delivered online using pre-recorded videos that someone will watch at home?

For example: the movement would not be practical if it involved movements that require people to turn their back to the computer screen for long periods of time, making it hard to view the instructor/video, and/or if the movements required too much room space

◯ Yes
◯ No

Section 10

Movement 10 Wave Hands Like Clouds

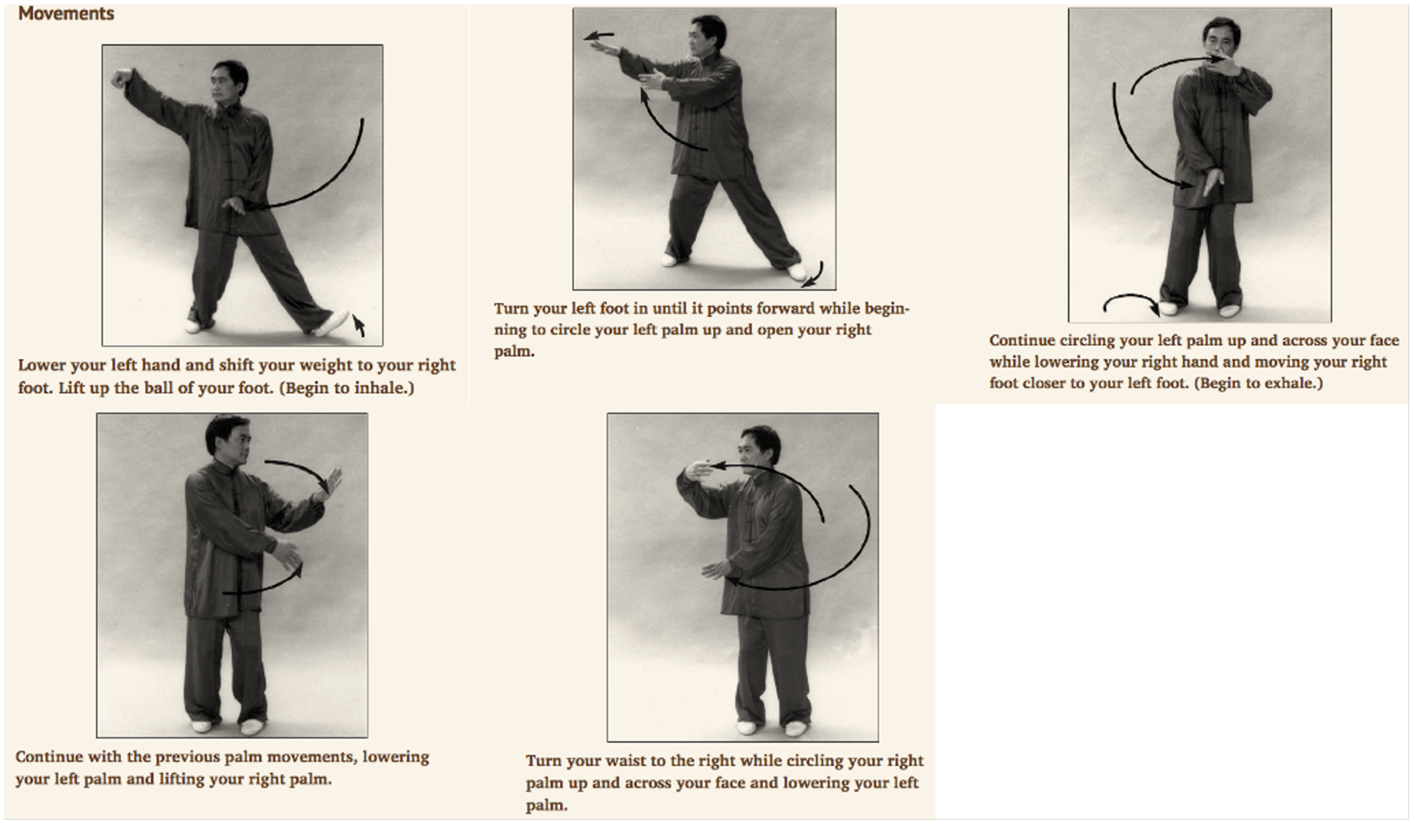

Is this movement appropriate for people with knee/hip osteoarthritis when done correctly?
Please select the one number on the scale that best represents your opinion with 0 = "Not at all appropriate for people with hip/knee OA" and 10 = "Completely appropriate for people with hip/knee OA when done correctly"

- ◯ 0 = Not at all appropriate for people with hip/knee OA
- ◯ 1
- ◯ 2
- ◯ 3
- ◯ 4
- ◯ 5
- ◯ 6
- ◯ 7
- ◯ 8
- ◯ 9
- ◯ 10 = Completely appropriate for people with hip/knee OA when done correctly

Is this movement safe to do unsupervised at home when done correctly?
Please select the one number on the scale that best represents your opinion with 0 = "Not at all safe for people to perform at home unsupervised" and 10= "Completely safe for people to perform at home unsupervised when done correctly"

- ◯ 0 = Not at all safe for people to perform at home unsupervised
- ◯ 1
- ◯ 2
- ◯ 3
- ◯ 4
- ◯ 5
- ◯ 6
- ◯ 7
- ◯ 8
- ◯ 9
- ◯ 10 = Completely safe for people to perform at home unsupervised when done correctly

Is this movement practical to be delivered online using pre-recorded videos that someone will watch at home?<br><br>For example: the movement would not be practical if it involved movements that require people to turn their back to the computer screen for long periods of time, making it hard to view the instructor/video, and/or if the movements required too much room space	◯ Yes<br>◯ No

Section 11

Movement 11 Single Whip

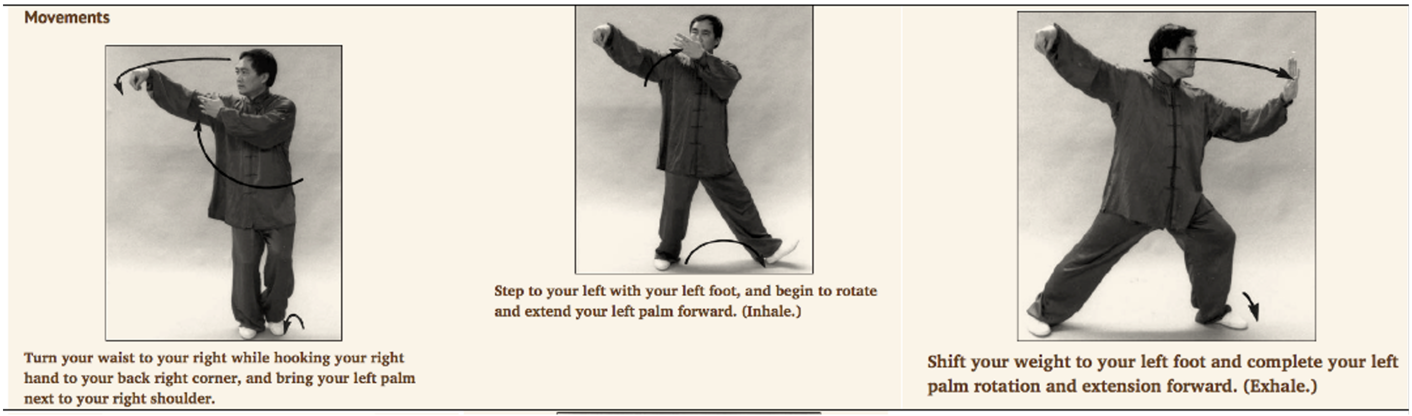

Is this movement appropriate for people with knee/hip osteoarthritis when done correctly?
Please select the one number on the scale that best represents your opinion with 0 = "Not at all appropriate for people with hip/knee OA" and 10 = "Completely appropriate for people with hip/knee OA when done correctly"

- ◯ 0 = Not at all appropriate for people with hip/knee OA
- ◯ 1
- ◯ 2
- ◯ 3
- ◯ 4
- ◯ 5
- ◯ 6
- ◯ 7
- ◯ 8
- ◯ 9
- ◯ 10 = Completely appropriate for people with hip/knee OA when done correctly

---

Is this movement safe to do unsupervised at home when done correctly?
Please select the one number on the scale that best represents your opinion with 0 = "Not at all safe for people to perform at home unsupervised" and 10= "Completely safe for people to perform at home unsupervised when done correctly"

- ◯ 0 = Not at all safe for people to perform at home unsupervised
- ◯ 1
- ◯ 2
- ◯ 3
- ◯ 4
- ◯ 5
- ◯ 6
- ◯ 7
- ◯ 8
- ◯ 9
- ◯ 10 = Completely safe for people to perform at home unsupervised when done correctly

---

Is this movement practical to be delivered online using pre-recorded videos that someone will watch at home?

For example: the movement would not be practical if it involved movements that require people to turn their back to the computer screen for long periods of time, making it hard to view the instructor/video, and/or if the movements required too much room space

- ◯ Yes
- ◯ No

Section 12

Movement 12 High Pat on Horse

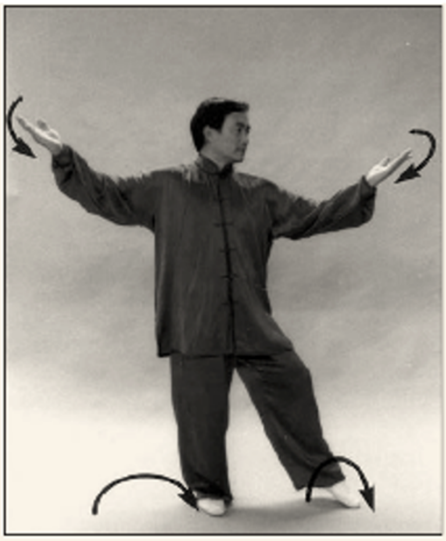

Bring your right foot a half step forward and shift all your weight on it while rotating both palms up. (Inhale.)

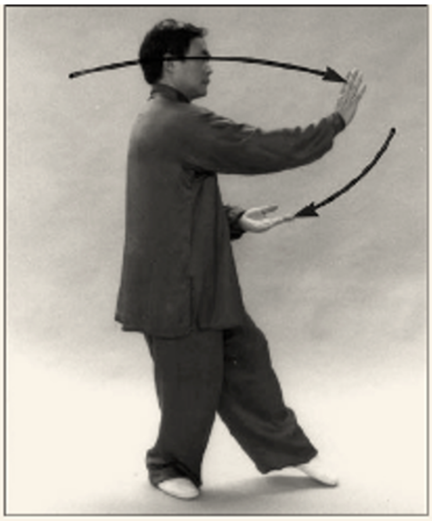

Bend your right elbow and extend your right palm forward while pulling your left palm next to your waist. (Exhale.)

Is this movement appropriate for people with knee/hip osteoarthritis when done correctly?
Please select the one number on the scale that best represents your opinion with 0 = "Not at all appropriate for people with hip/knee OA" and 10 = "Completely appropriate for people with hip/knee OA when done correctly"

- ◯ 0 = Not at all appropriate for people with hip/knee OA
- ◯ 1
- ◯ 2
- ◯ 3
- ◯ 4
- ◯ 5
- ◯ 6
- ◯ 7
- ◯ 8
- ◯ 9
- ◯ 10 = Completely appropriate for people with hip/knee OA when done correctly

Is this movement safe to do unsupervised at home when done correctly?
Please select the one number on the scale that best represents your opinion with 0 = "Not at all safe for people to perform at home unsupervised" and 10= "Completely safe for people to perform at home unsupervised when done correctly"

- ◯ 0 = Not at all safe for people to perform at home unsupervised
- ◯ 1
- ◯ 2
- ◯ 3
- ◯ 4
- ◯ 5
- ◯ 6
- ◯ 7
- ◯ 8
- ◯ 9
- ◯ 10 = Completely safe for people to perform at home unsupervised when done correctly

Is this movement practical to be delivered online using pre-recorded videos that someone will watch at home?

For example: the movement would not be practical if it involved movements that require people to turn their back to the computer screen for long periods of time, making it hard to view the instructor/video, and/or if the movements required too much room space

- ◯ Yes
- ◯ No

Section 13

Movement 13 Right Heel Kick

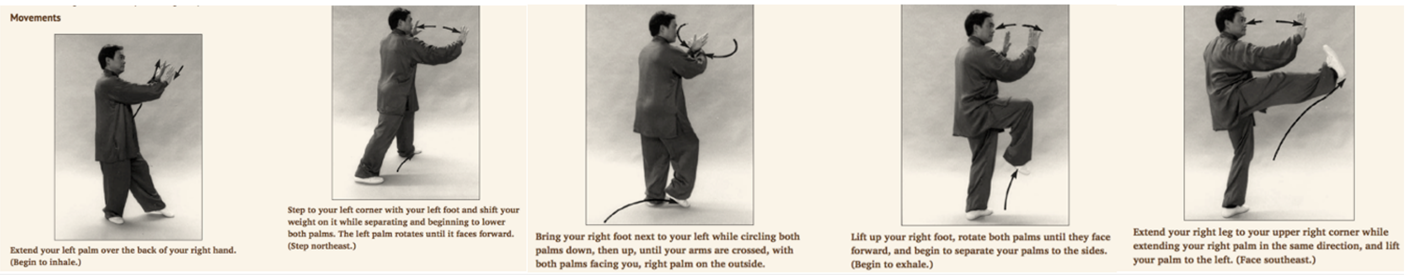

Is this movement appropriate for people with knee/hip osteoarthritis when done correctly?
Please select the one number on the scale that best represents your opinion with 0 = "Not at all appropriate for people with hip/knee OA" and 10 = "Completely appropriate for people with hip/knee OA when done correctly"

- ◯ 0 = Not at all appropriate for people with hip/knee OA
- ◯ 1
- ◯ 2
- ◯ 3
- ◯ 4
- ◯ 5
- ◯ 6
- ◯ 7
- ◯ 8
- ◯ 9
- ◯ 10 = Completely appropriate for people with hip/knee OA when done correctly

---

Is this movement safe to do unsupervised at home when done correctly?
Please select the one number on the scale that best represents your opinion with 0 = "Not at all safe for people to perform at home unsupervised" and 10= "Completely safe for people to perform at home unsupervised when done correctly"

- ◯ 0 = Not at all safe for people to perform at home unsupervised
- ◯ 1
- ◯ 2
- ◯ 3
- ◯ 4
- ◯ 5
- ◯ 6
- ◯ 7
- ◯ 8
- ◯ 9
- ◯ 10 = Completely safe for people to perform at home unsupervised when done correctly

---

Is this movement practical to be delivered online using pre-recorded videos that someone will watch at home?

For example: the movement would not be practical if it involved movements that require people to turn their back to the computer screen for long periods of time, making it hard to view the instructor/video, and/or if the movements required too much room space

- ◯ Yes
- ◯ No

Section 14

Movement 13 Modified- Right Heel Kick

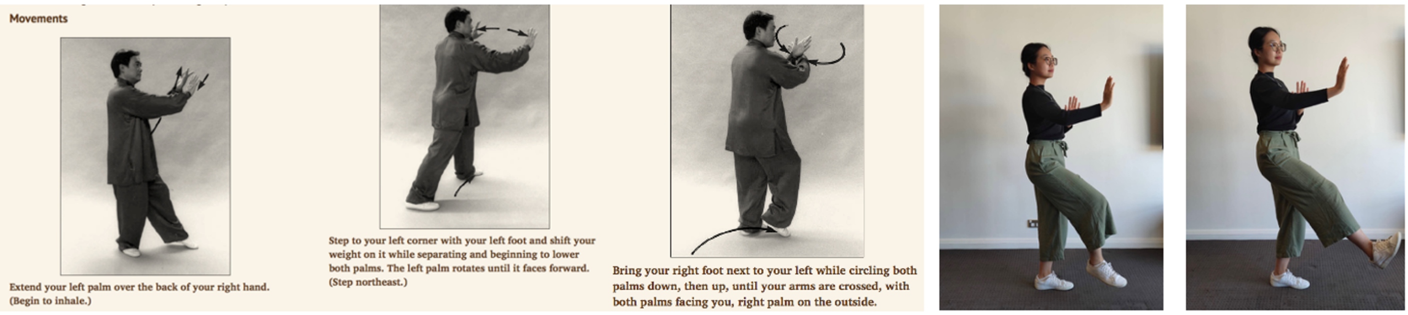

Is this movement appropriate for people with knee/hip osteoarthritis when done correctly?<br>Please select the one number on the scale that best represents your opinion with 0 = "Not at all appropriate for people with hip/knee OA" and 10 = "Completely appropriate for people with hip/knee OA when done correctly"	◯ 0 = Not at all appropriate for people with hip/knee OA<br>◯ 1<br>◯ 2<br>◯ 3<br>◯ 4<br>◯ 5<br>◯ 6<br>◯ 7<br>◯ 8<br>◯ 9<br>◯ 10 = Completely appropriate for people with hip/knee OA when done correctly
Is this movement safe to do unsupervised at home when done correctly?<br>Please select the one number on the scale that best represents your opinion with 0 = "Not at all safe for people to perform at home unsupervised" and 10= "Completely safe for people to perform at home unsupervised when done correctly"	◯ 0 = Not at all safe for people to perform at home unsupervised<br>◯ 1<br>◯ 2<br>◯ 3<br>◯ 4<br>◯ 5<br>◯ 6<br>◯ 7<br>◯ 8<br>◯ 9<br>◯ 10 = Completely safe for people to perform at home unsupervised when done correctly
Is this movement practical to be delivered online using pre-recorded videos that someone will watch at home?<br><br>For example: the movement would not be practical if it involved movements that require people to turn their back to the computer screen for long periods of time, making it hard to view the instructor/video, and/or if the movements required too much room space	◯ Yes<br>◯ No

Section 15

Movement 14 Strike to the Ears with Both Fists

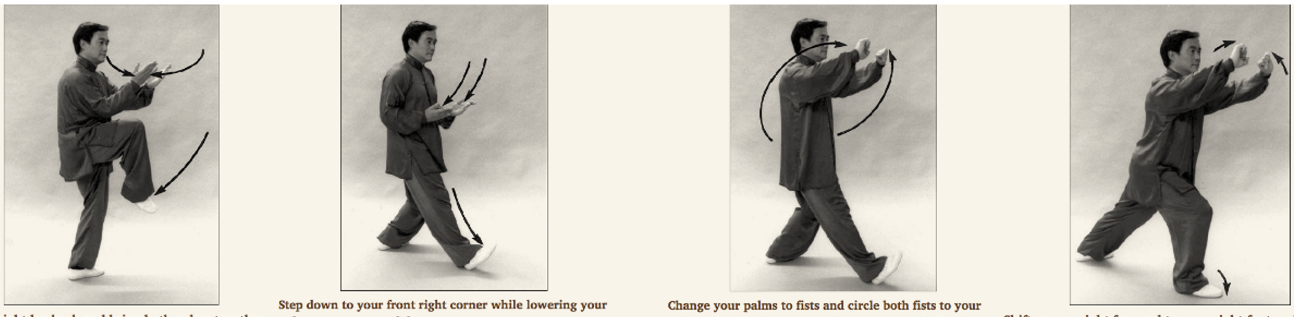

Pull your right leg back and bring both palms together until your palms are facing you. (Begin to inhale.)

Step down to your front right corner while lowering your palms next to your abdomen.

Change your palms to fists and circle both fists to your sides and up. Palms of your fists face forward. (Begin to exhale.)

Shift your weight forward to your right foot and bring your fists closer together. (Face southeast.)

Is this movement appropriate for people with knee/hip osteoarthritis when done correctly?
Please select the one number on the scale that best represents your opinion with 0 = "Not at all appropriate for people with hip/knee OA" and 10 = "Completely appropriate for people with hip/knee OA when done correctly"

- ◯ 0 = Not at all appropriate for people with hip/knee OA
- ◯ 1
- ◯ 2
- ◯ 3
- ◯ 4
- ◯ 5
- ◯ 6
- ◯ 7
- ◯ 8
- ◯ 9
- ◯ 10 = Completely appropriate for people with hip/knee OA when done correctly

---

Is this movement safe to do unsupervised at home when done correctly?
Please select the one number on the scale that best represents your opinion with 0 = "Not at all safe for people to perform at home unsupervised" and 10= "Completely safe for people to perform at home unsupervised when done correctly"

- ◯ 0 = Not at all safe for people to perform at home unsupervised
- ◯ 1
- ◯ 2
- ◯ 3
- ◯ 4
- ◯ 5
- ◯ 6
- ◯ 7
- ◯ 8
- ◯ 9
- ◯ 10 = Completely safe for people to perform at home unsupervised when done correctly

---

Is this movement practical to be delivered online using pre-recorded videos that someone will watch at home?

For example: the movement would not be practical if it involved movements that require people to turn their back to the computer screen for long periods of time, making it hard to view the instructor/video, and/or if the movements required too much room space

- ◯ Yes
- ◯ No

Section 16

Movement 15 Turn and Left Heel Kick

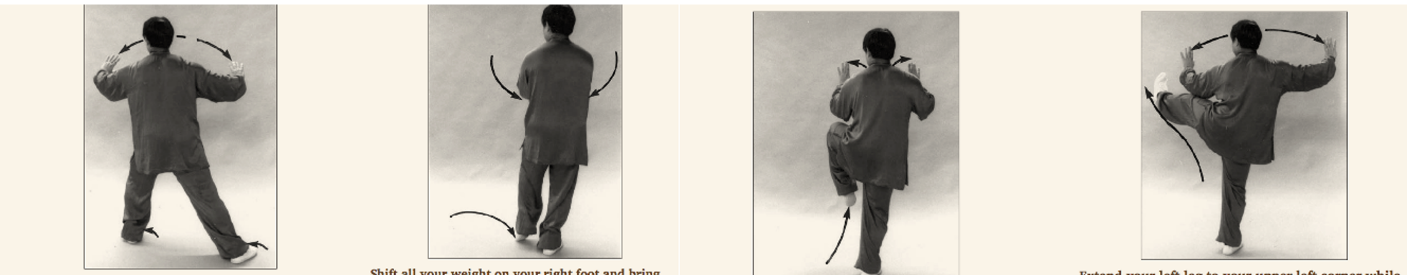

Turn your right foot in and left foot out as you turn to your left. Shift your weight to your left foot. Open your fists and separate your palms to your sides. (Begin to inhale.)

Shift all your weight on your right foot and bring your left foot next to your right while circling both palms down until they cross and the palms face you, left palm on the outside. (Similar to palm movements in posture 13.)

Lift up your left foot, rotate both palms until they face forward, and begin to separate your palms to the sides. (Begin to exhale.)

Extend your left leg to your upper left corner while extending your left palm in the same direction and right palm to your right. (Kick northwest.)

Is this movement appropriate for people with knee/hip osteoarthritis when done correctly?
Please select the one number on the scale that best represents your opinion with 0 = "Not at all appropriate for people with hip/knee OA" and 10 = "Completely appropriate for people with hip/knee OA when done correctly"

- ◯ 0 = Not at all appropriate for people with hip/knee OA
- ◯ 1
- ◯ 2
- ◯ 3
- ◯ 4
- ◯ 5
- ◯ 6
- ◯ 7
- ◯ 8
- ◯ 9
- ◯ 10 = Completely appropriate for people with hip/knee OA when done correctly

---

Is this movement safe to do unsupervised at home when done correctly?
Please select the one number on the scale that best represents your opinion with 0 = "Not at all safe for people to perform at home unsupervised" and 10= "Completely safe for people to perform at home unsupervised when done correctly"

- ◯ 0 = Not at all safe for people to perform at home unsupervised
- ◯ 1
- ◯ 2
- ◯ 3
- ◯ 4
- ◯ 5
- ◯ 6
- ◯ 7
- ◯ 8
- ◯ 9
- ◯ 10 = Completely safe for people to perform at home unsupervised when done correctly

---

Is this movement practical to be delivered online using pre-recorded videos that someone will watch at home?

For example: the movement would not be practical if it involved movements that require people to turn their back to the computer screen for long periods of time, making it hard to view the instructor/video, and/or if the movements required too much room space

- ◯ Yes
- ◯ No

Section 17

Movement 15 Modified- Left Heel Kick

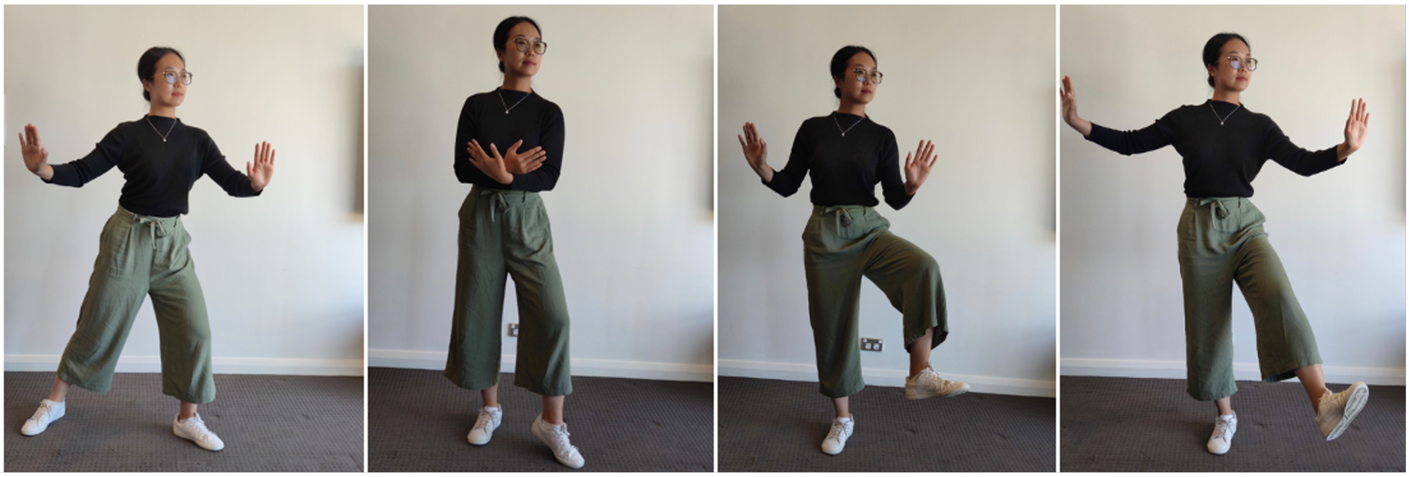

Is this movement appropriate for people with knee/hip osteoarthritis when done correctly?<br>Please select the one number on the scale that best represents your opinion with 0 = "Not at all appropriate for people with hip/knee OA" and 10 = "Completely appropriate for people with hip/knee OA when done correctly"	◯ 0 = Not at all appropriate for people with hip/knee OA<br>◯ 1<br>◯ 2<br>◯ 3<br>◯ 4<br>◯ 5<br>◯ 6<br>◯ 7<br>◯ 8<br>◯ 9<br>◯ 10 = Completely appropriate for people with hip/knee OA when done correctly
Is this movement safe to do unsupervised at home when done correctly?<br>Please select the one number on the scale that best represents your opinion with 0 = "Not at all safe for people to perform at home unsupervised" and 10= "Completely safe for people to perform at home unsupervised when done correctly"	◯ 0 = Not at all safe for people to perform at home unsupervised<br>◯ 1<br>◯ 2<br>◯ 3<br>◯ 4<br>◯ 5<br>◯ 6<br>◯ 7<br>◯ 8<br>◯ 9<br>◯ 10 = Completely safe for people to perform at home unsupervised when done correctly
Is this movement practical to be delivered online using pre-recorded videos that someone will watch at home?<br><br>For example: the movement would not be practical if it involved movements that require people to turn their back to the computer screen for long periods of time, making it hard to view the instructor/video, and/or if the movements required too much room space	◯ Yes<br>◯ No

Section 18

Movement 16a Left Lower Body/ Snake Creeps Down/ Single Whip Squatting Down

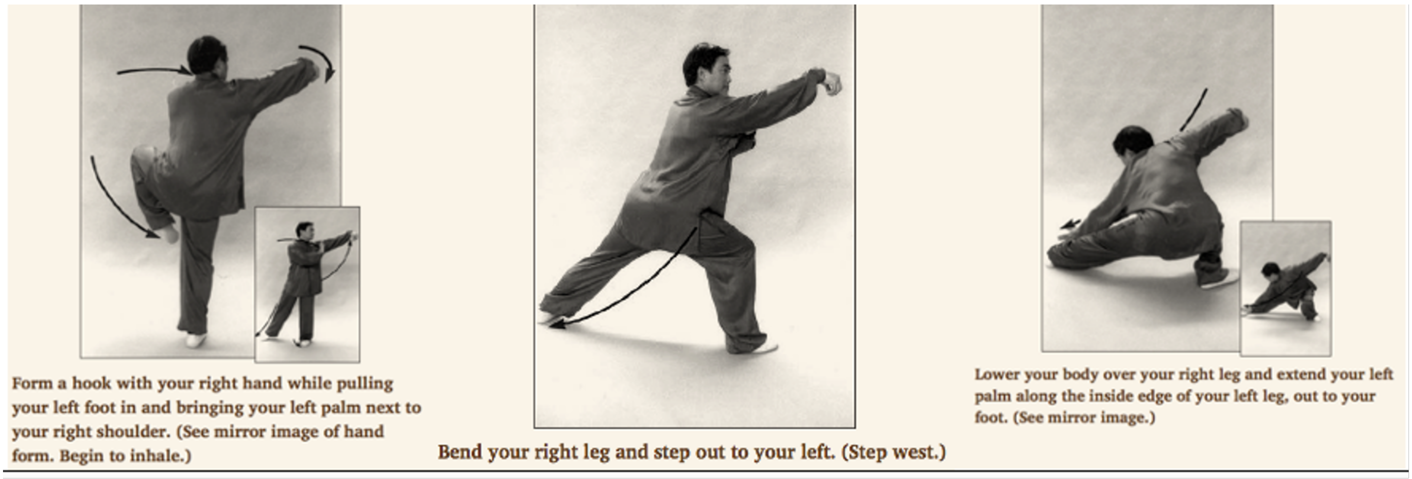

Is this movement appropriate for people with knee/hip osteoarthritis when done correctly?
Please select the one number on the scale that best represents your opinion with 0 = "Not at all appropriate for people with hip/knee OA" and 10 = "Completely appropriate for people with hip/knee OA when done correctly"

- ◯ 0 = Not at all appropriate for people with hip/knee OA
- ◯ 1
- ◯ 2
- ◯ 3
- ◯ 4
- ◯ 5
- ◯ 6
- ◯ 7
- ◯ 8
- ◯ 9
- ◯ 10 = Completely appropriate for people with hip/knee OA when done correctly

---

Is this movement safe to do unsupervised at home when done correctly?
Please select the one number on the scale that best represents your opinion with 0 = "Not at all safe for people to perform at home unsupervised" and 10= "Completely safe for people to perform at home unsupervised when done correctly"

- ◯ 0 = Not at all safe for people to perform at home unsupervised
- ◯ 1
- ◯ 2
- ◯ 3
- ◯ 4
- ◯ 5
- ◯ 6
- ◯ 7
- ◯ 8
- ◯ 9
- ◯ 10 = Completely safe for people to perform at home unsupervised when done correctly

---

Is this movement practical to be delivered online using pre-recorded videos that someone will watch at home?

For example: the movement would not be practical if it involved movements that require people to turn their back to the computer screen for long periods of time, making it hard to view the instructor/video, and/or if the movements required too much room space

- ◯ Yes
- ◯ No

Section 19

Movement 16a Modified- Left Lower Body/ Snake Creeps Down/ Single Whip Squatting Down

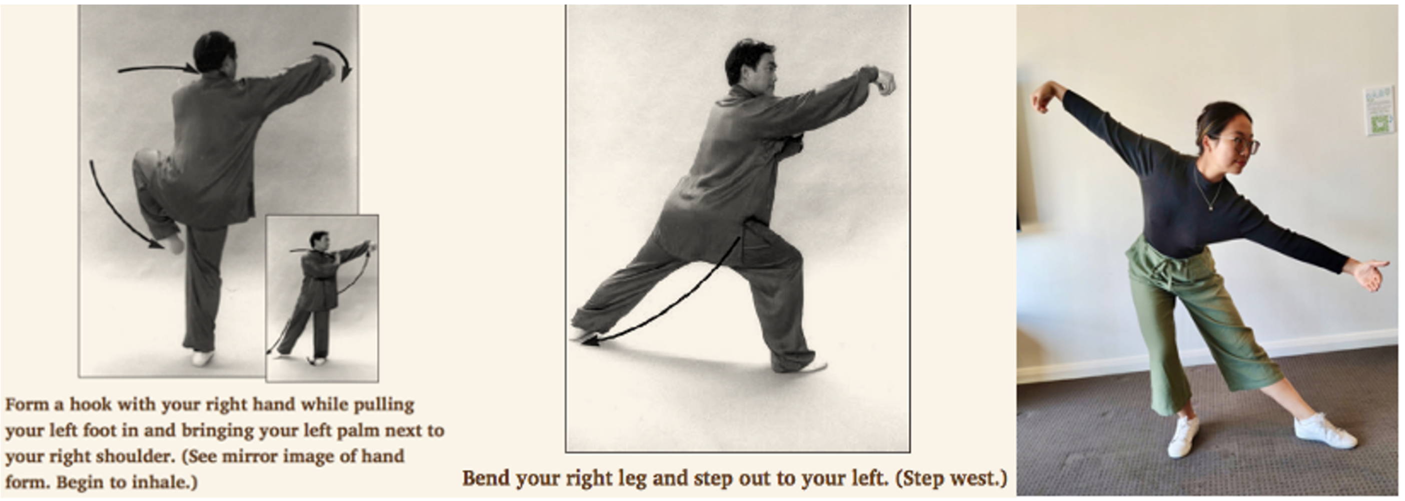

Is this movement appropriate for people with knee/hip osteoarthritis when done correctly?
Please select the one number on the scale that best represents your opinion with 0 = "Not at all appropriate for people with hip/knee OA" and 10 = "Completely appropriate for people with hip/knee OA when done correctly"

- ◯ 0 = Not at all appropriate for people with hip/knee OA
- ◯ 1
- ◯ 2
- ◯ 3
- ◯ 4
- ◯ 5
- ◯ 6
- ◯ 7
- ◯ 8
- ◯ 9
- ◯ 10 = Completely appropriate for people with hip/knee OA when done correctly

---

Is this movement safe to do unsupervised at home when done correctly?
Please select the one number on the scale that best represents your opinion with 0 = "Not at all safe for people to perform at home unsupervised" and 10= "Completely safe for people to perform at home unsupervised when done correctly"

- ◯ 0 = Not at all safe for people to perform at home unsupervised
- ◯ 1
- ◯ 2
- ◯ 3
- ◯ 4
- ◯ 5
- ◯ 6
- ◯ 7
- ◯ 8
- ◯ 9
- ◯ 10 = Completely safe for people to perform at home unsupervised when done correctly

---

Is this movement practical to be delivered online using pre-recorded videos that someone will watch at home?

For example: the movement would not be practical if it involved movements that require people to turn their back to the computer screen for long periods of time, making it hard to view the instructor/video, and/or if the movements required too much room space

- ◯ Yes
- ◯ No

Section 20

Movement 16b - Golden Rooster /Stands on One Leg

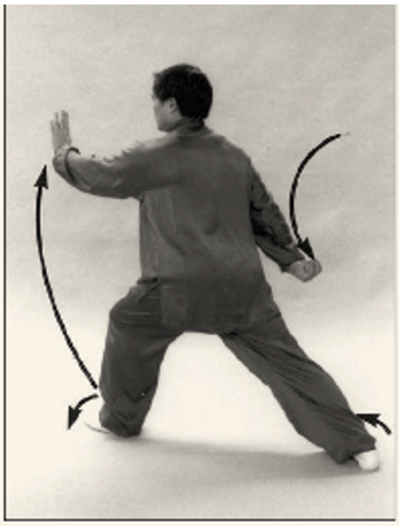

Turn your left foot until it points forward. Shift your weight forward into left bow stance while lifting your left palm up and forward and lowering your right hand behind you in a hook position, pointing up. (Face west. Begin to exhale.)

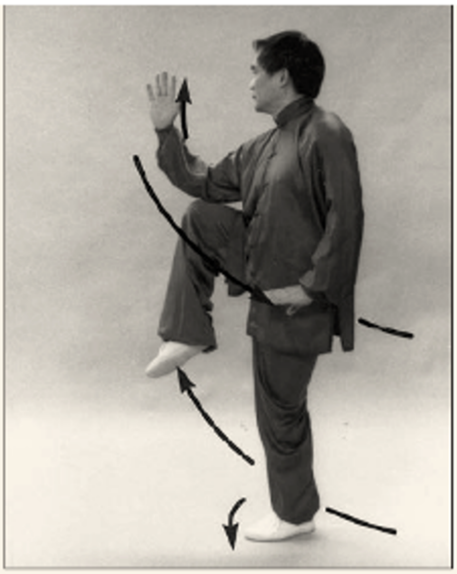

Turn your left foot out and stand up on it while lowering your left palm. At the same time, open your right hand and spear up.

Is this movement appropriate for people with knee/hip osteoarthritis when done correctly?
Please select the one number on the scale that best represents your opinion with 0 = "Not at all appropriate for people with hip/knee OA" and 10 = "Completely appropriate for people with hip/knee OA when done correctly"

- ◯ 0 = Not at all appropriate for people with hip/knee OA
- ◯ 1
- ◯ 2
- ◯ 3
- ◯ 4
- ◯ 5
- ◯ 6
- ◯ 7
- ◯ 8
- ◯ 9
- ◯ 10 = Completely appropriate for people with hip/knee OA when done correctly

Is this movement safe to do unsupervised at home when done correctly?
Please select the one number on the scale that best represents your opinion with 0 = "Not at all safe for people to perform at home unsupervised" and 10= "Completely safe for people to perform at home unsupervised when done correctly"

- ◯ 0 = Not at all safe for people to perform at home unsupervised
- ◯ 1
- ◯ 2
- ◯ 3
- ◯ 4
- ◯ 5
- ◯ 6
- ◯ 7
- ◯ 8
- ◯ 9
- ◯ 10 = Completely safe for people to perform at home unsupervised when done correctly

Is this movement practical to be delivered online using pre-recorded videos that someone will watch at home?

For example: the movement would not be practical if it involved movements that require people to turn their back to the computer screen for long periods of time, making it hard to view the instructor/video, and/or if the movements required too much room space

◯ Yes
◯ No

Section 21

Movement 16b Modified - Golden Rooster /Stands on One Leg

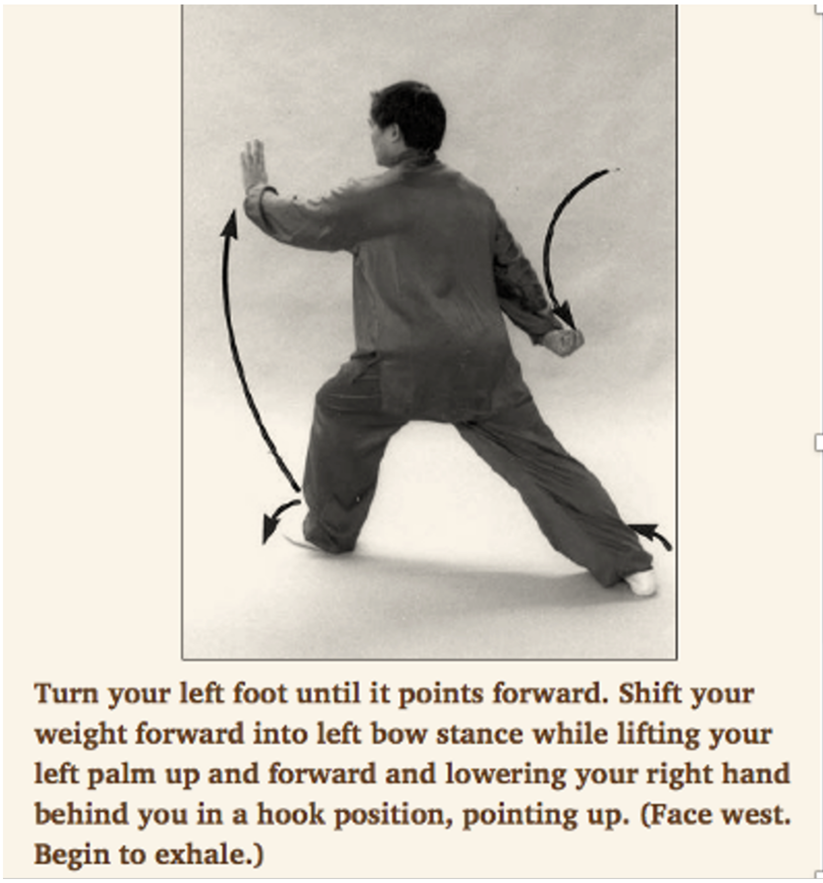

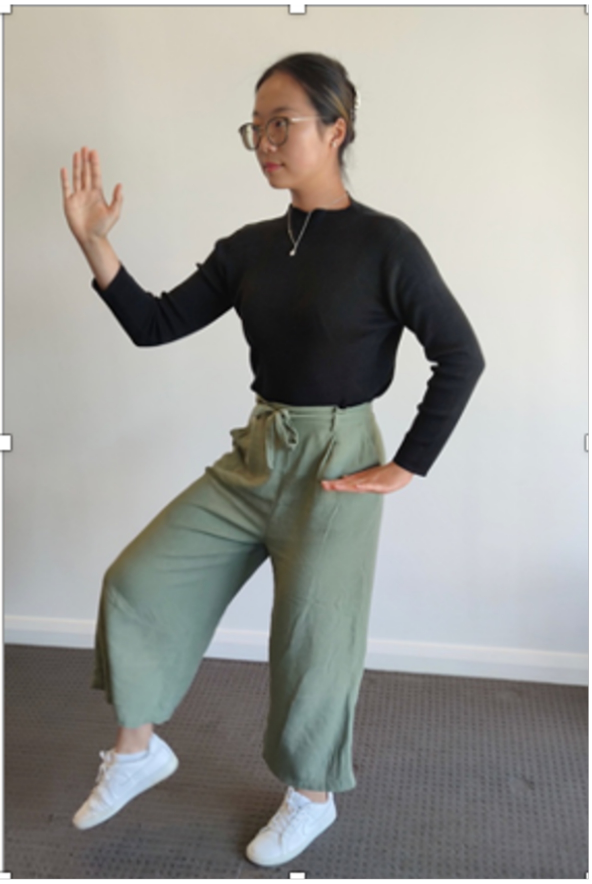

Is this movement appropriate for people with knee/hip osteoarthritis when done correctly?
Please select the one number on the scale that best represents your opinion with 0 = "Not at all appropriate for people with hip/knee OA" and 10 = "Completely appropriate for people with hip/knee OA when done correctly"

- ◯ 0 = Not at all appropriate for people with hip/knee OA
- ◯ 1
- ◯ 2
- ◯ 3
- ◯ 4
- ◯ 5
- ◯ 6
- ◯ 7
- ◯ 8
- ◯ 9
- ◯ 10 = Completely appropriate for people with hip/knee OA when done correctly

---

Is this movement safe to do unsupervised at home when done correctly?
Please select the one number on the scale that best represents your opinion with 0 = "Not at all safe for people to perform at home unsupervised" and 10= "Completely safe for people to perform at home unsupervised when done correctly"

- ◯ 0 = Not at all safe for people to perform at home unsupervised
- ◯ 1
- ◯ 2
- ◯ 3
- ◯ 4
- ◯ 5
- ◯ 6
- ◯ 7
- ◯ 8
- ◯ 9
- ◯ 10 = Completely safe for people to perform at home unsupervised when done correctly

Is this movement practical to be delivered online using pre-recorded videos that someone will watch at home?<br><br>For example: the movement would not be practical if it involved movements that require people to turn their back to the computer screen for long periods of time, making it hard to view the instructor/video, and/or if the movements required too much room space	◯ Yes<br>◯ No

Section 22

Movement 17a Right Lower Body/ Snake Creeps Down/ Single Whip Squatting Down

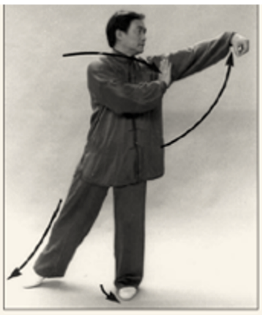

Put your right foot down in front of you. Form a hook with your left hand, and then lift and extend it backward while placing your right palm next to your left shoulder. Turn your left foot out to get ready for the next move. (Begin to inhale.)

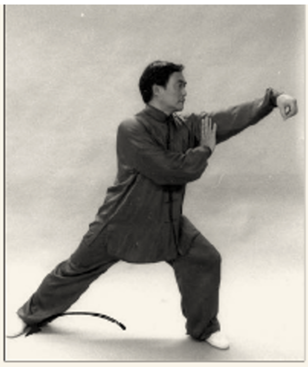

Step to your right with your right foot and bend your left knee. (Step west.)

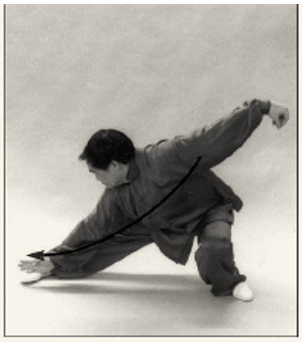

Lower your body over your left leg and extend your right palm along the inside edge of your right leg, out to your foot.

Is this movement appropriate for people with knee/hip osteoarthritis when done correctly?
Please select the one number on the scale that best represents your opinion with 0 = "Not at all appropriate for people with hip/knee OA" and 10 = "Completely appropriate for people with hip/knee OA when done correctly"

- ◯ 0 = Not at all appropriate for people with hip/knee OA
- ◯ 1
- ◯ 2
- ◯ 3
- ◯ 4
- ◯ 5
- ◯ 6
- ◯ 7
- ◯ 8
- ◯ 9
- ◯ 10 = Completely appropriate for people with hip/knee OA when done correctly

---

Is this movement safe to do unsupervised at home when done correctly?
Please select the one number on the scale that best represents your opinion with 0 = "Not at all safe for people to perform at home unsupervised" and 10= "Completely safe for people to perform at home unsupervised when done correctly"

- ◯ 0 = Not at all safe for people to perform at home unsupervised
- ◯ 1
- ◯ 2
- ◯ 3
- ◯ 4
- ◯ 5
- ◯ 6
- ◯ 7
- ◯ 8
- ◯ 9
- ◯ 10 = Completely safe for people to perform at home unsupervised when done correctly

---

Is this movement practical to be delivered online using pre-recorded videos that someone will watch at home?

For example: the movement would not be practical if it involved movements that require people to turn their back to the computer screen for long periods of time, making it hard to view the instructor/video, and/or if the movements required too much room space

- ◯ Yes
- ◯ No

Section 23

Movement 17a Modified- Right Lower Body/ Snake Creeps Down/ Single Whip Squatting Down

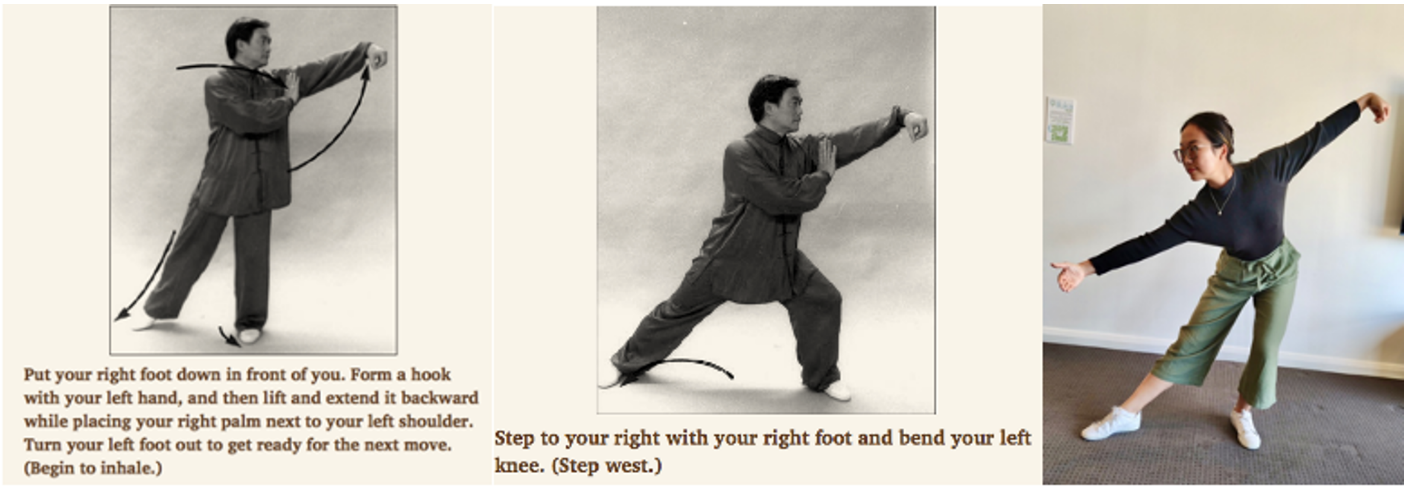

Is this movement appropriate for people with knee/hip osteoarthritis when done correctly?
Please select the one number on the scale that best represents your opinion with 0 = "Not at all appropriate for people with hip/knee OA" and 10 = "Completely appropriate for people with hip/knee OA when done correctly"

- ◯ 0 = Not at all appropriate for people with hip/knee OA
- ◯ 1
- ◯ 2
- ◯ 3
- ◯ 4
- ◯ 5
- ◯ 6
- ◯ 7
- ◯ 8
- ◯ 9
- ◯ 10 = Completely appropriate for people with hip/knee OA when done correctly

Is this movement safe to do unsupervised at home when done correctly?
Please select the one number on the scale that best represents your opinion with 0 = "Not at all safe for people to perform at home unsupervised" and 10= "Completely safe for people to perform at home unsupervised when done correctly"

- ◯ 0 = Not at all safe for people to perform at home unsupervised
- ◯ 1
- ◯ 2
- ◯ 3
- ◯ 4
- ◯ 5
- ◯ 6
- ◯ 7
- ◯ 8
- ◯ 9
- ◯ 10 = Completely safe for people to perform at home unsupervised when done correctly

Is this movement practical to be delivered online using pre-recorded videos that someone will watch at home?

For example: the movement would not be practical if it involved movements that require people to turn their back to the computer screen for long periods of time, making it hard to view the instructor/video, and/or if the movements required too much room space

- ◯ Yes
- ◯ No

Section 24

Movement 17b - Golden Rooster /Stands on One Leg

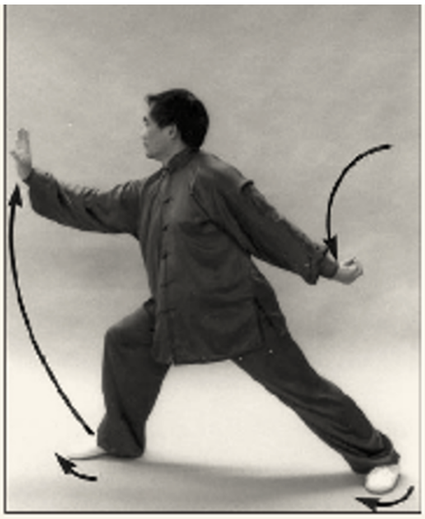

Turn your right foot until it points forward. Shift your weight forward into right bow stance while lifting your right palm and lowering your left hand behind you in a hook position, pointing up. (Face west. Begin to exhale.)

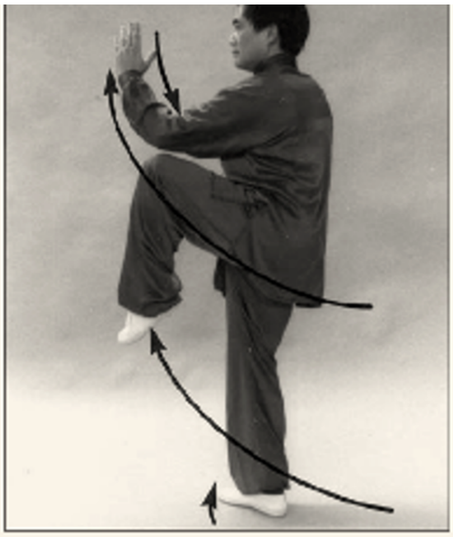

Turn your right foot out and stand up on it while lowering your right palm. At the same time, open your left hand and spear up.

Is this movement appropriate for people with knee/hip osteoarthritis when done correctly?<br>Please select the one number on the scale that best represents your opinion with 0 = "Not at all appropriate for people with hip/knee OA" and 10 = "Completely appropriate for people with hip/knee OA when done correctly"	◯ 0 = Not at all appropriate for people with hip/knee OA<br>◯ 1<br>◯ 2<br>◯ 3<br>◯ 4<br>◯ 5<br>◯ 6<br>◯ 7<br>◯ 8<br>◯ 9<br>◯ 10 = Completely appropriate for people with hip/knee OA when done correctly
Is this movement safe to do unsupervised at home when done correctly?<br>Please select the one number on the scale that best represents your opinion with 0 = "Not at all safe for people to perform at home unsupervised" and 10= "Completely safe for people to perform at home unsupervised when done correctly"	◯ 0 = Not at all safe for people to perform at home unsupervised<br>◯ 1<br>◯ 2<br>◯ 3<br>◯ 4<br>◯ 5<br>◯ 6<br>◯ 7<br>◯ 8<br>◯ 9<br>◯ 10 = Completely safe for people to perform at home unsupervised when done correctly

Is this movement practical to be delivered online using pre-recorded videos that someone will watch at home?<br><br>For example: the movement would not be practical if it involved movements that require people to turn their back to the computer screen for long periods of time, making it hard to view the instructor/video, and/or if the movements required too much room space	◯ Yes<br>◯ No

Section 25

Movement 17b Modified - Golden Rooster/ Stands on One Leg

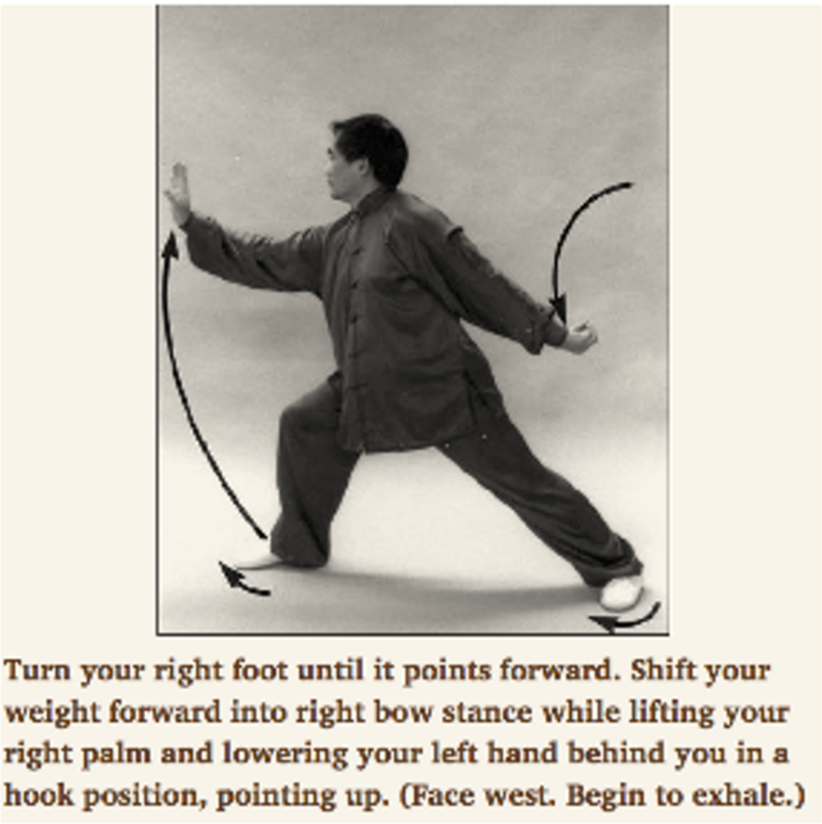

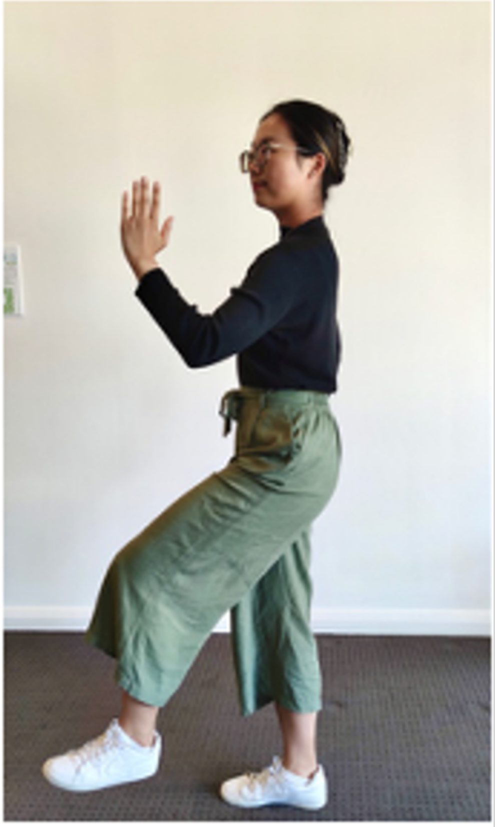

Is this movement appropriate for people with knee/hip osteoarthritis when done correctly?
Please select the one number on the scale that best represents your opinion with 0 = "Not at all appropriate for people with hip/knee OA" and 10 = "Completely appropriate for people with hip/knee OA when done correctly"

- ◯ 0 = Not at all appropriate for people with hip/knee OA
- ◯ 1
- ◯ 2
- ◯ 3
- ◯ 4
- ◯ 5
- ◯ 6
- ◯ 7
- ◯ 8
- ◯ 9
- ◯ 10 = Completely appropriate for people with hip/knee OA when done correctly

Is this movement safe to do unsupervised at home when done correctly?
Please select the one number on the scale that best represents your opinion with 0 = "Not at all safe for people to perform at home unsupervised" and 10= "Completely safe for people to perform at home unsupervised when done correctly"

- ◯ 0 = Not at all safe for people to perform at home unsupervised
- ◯ 1
- ◯ 2
- ◯ 3
- ◯ 4
- ◯ 5
- ◯ 6
- ◯ 7
- ◯ 8
- ◯ 9
- ◯ 10 = Completely safe for people to perform at home unsupervised when done correctly

Is this movement practical to be delivered online using pre-recorded videos that someone will watch at home?

For example: the movement would not be practical if it involved movements that require people to turn their back to the computer screen for long periods of time, making it hard to view the instructor/video, and/or if the movements required too much room space

◯ Yes
◯ No

Section 26

Movement 18 Fair Lady Works with Shuttles/ Jade Lady, Ride side, Left side

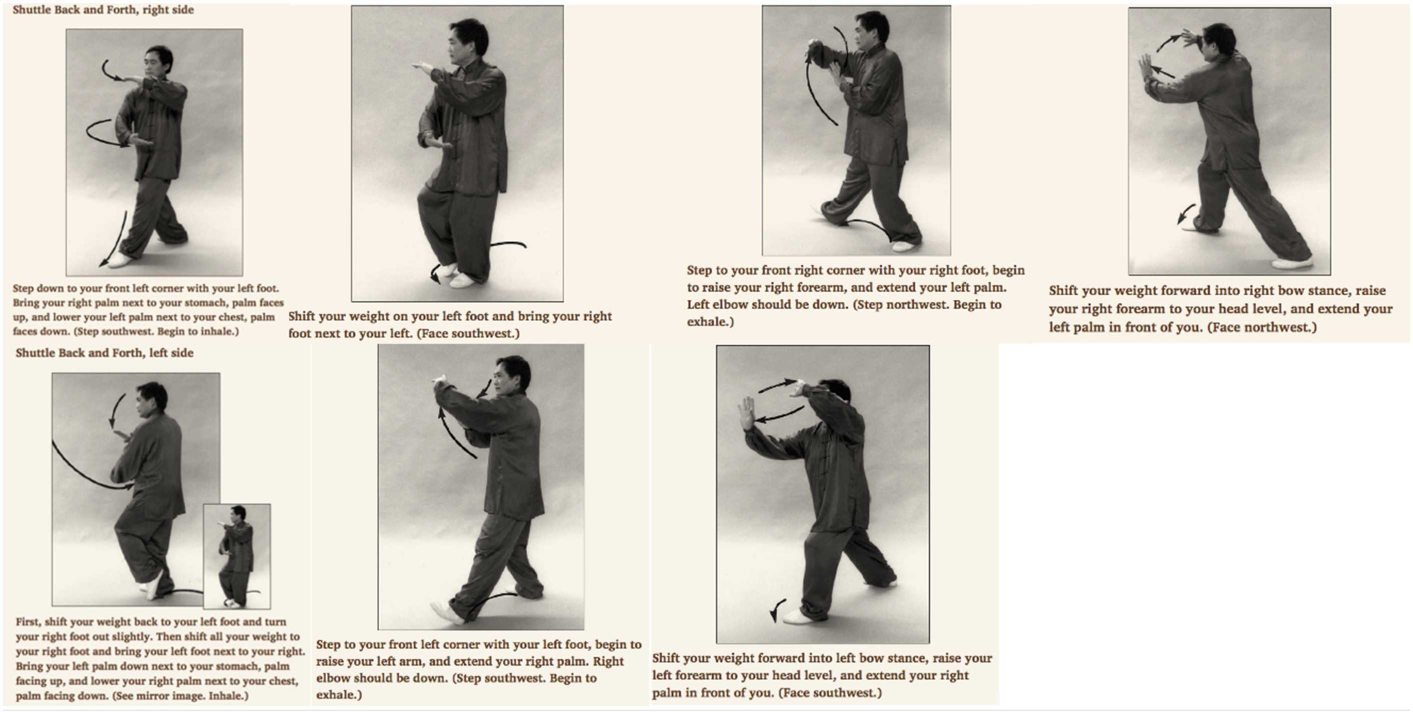

Is this movement appropriate for people with knee/hip osteoarthritis when done correctly?
Please select the one number on the scale that best represents your opinion with 0 = "Not at all appropriate for people with hip/knee OA" and 10 = "Completely appropriate for people with hip/knee OA when done correctly"

- ◯ 0 = Not at all appropriate for people with hip/knee OA
- ◯ 1
- ◯ 2
- ◯ 3
- ◯ 4
- ◯ 5
- ◯ 6
- ◯ 7
- ◯ 8
- ◯ 9
- ◯ 10 = Completely appropriate for people with hip/knee OA when done correctly

Is this movement safe to do unsupervised at home when done correctly?
Please select the one number on the scale that best represents your opinion with 0 = "Not at all safe for people to perform at home unsupervised" and 10= "Completely safe for people to perform at home unsupervised when done correctly"

- ◯ 0 = Not at all safe for people to perform at home unsupervised
- ◯ 1
- ◯ 2
- ◯ 3
- ◯ 4
- ◯ 5
- ◯ 6
- ◯ 7
- ◯ 8
- ◯ 9
- ◯ 10 = Completely safe for people to perform at home unsupervised when done correctly

Is this movement practical to be delivered online using pre-recorded videos that someone will watch at home?<br><br>For example: the movement would not be practical if it involved movements that require people to turn their back to the computer screen for long periods of time, making it hard to view the instructor/video, and/or if the movements required too much room space	◯ Yes<br>◯ No

Section 27

Movement 19 Needle at Sea Bottom

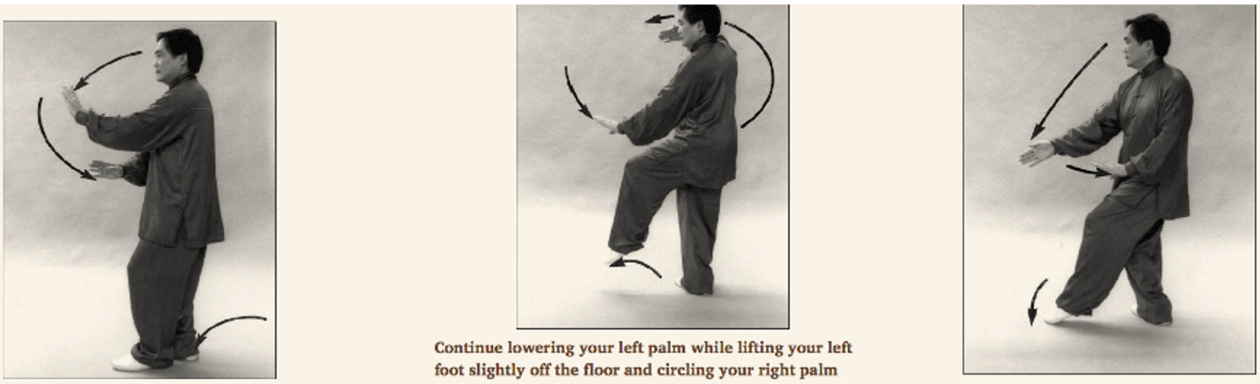

Bring your right foot behind your left and shift all your weight on it, and begin lowering both palms in front of you. (Face west. Inhale.)

Continue lowering your left palm while lifting your left foot slightly off the floor and circling your right palm back and up until it is next to your ear. (Begin to exhale.)

Pull your left palm next to your waist, touch down on your left foot, and spear down with your right palm. (Face west.)

Is this movement appropriate for people with knee/hip osteoarthritis when done correctly?
Please select the one number on the scale that best represents your opinion with 0 = "Not at all appropriate for people with hip/knee OA" and 10 = "Completely appropriate for people with hip/knee OA when done correctly"

- ◯ 0 = Not at all appropriate for people with hip/knee OA
- ◯ 1
- ◯ 2
- ◯ 3
- ◯ 4
- ◯ 5
- ◯ 6
- ◯ 7
- ◯ 8
- ◯ 9
- ◯ 10 = Completely appropriate for people with hip/knee OA when done correctly

---

Is this movement safe to do unsupervised at home when done correctly?
Please select the one number on the scale that best represents your opinion with 0 = "Not at all safe for people to perform at home unsupervised" and 10= "Completely safe for people to perform at home unsupervised when done correctly"

- ◯ 0 = Not at all safe for people to perform at home unsupervised
- ◯ 1
- ◯ 2
- ◯ 3
- ◯ 4
- ◯ 5
- ◯ 6
- ◯ 7
- ◯ 8
- ◯ 9
- ◯ 10 = Completely safe for people to perform at home unsupervised when done correctly

---

Is this movement practical to be delivered online using pre-recorded videos that someone will watch at home?

For example: the movement would not be practical if it involved movements that require people to turn their back to the computer screen for long periods of time, making it hard to view the instructor/video, and/or if the movements required too much room space

- ◯ Yes
- ◯ No

Section 28

Movement 20 Fan Through Back/ Flash Arms

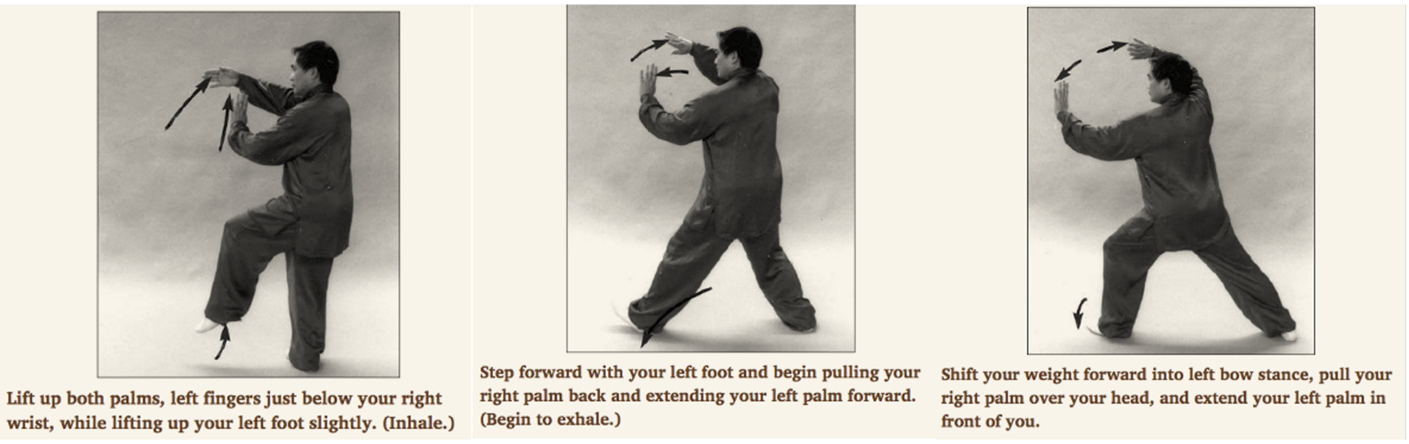

Is this movement appropriate for people with knee/hip osteoarthritis when done correctly?
Please select the one number on the scale that best represents your opinion with 0 = "Not at all appropriate for people with hip/knee OA" and 10 = "Completely appropriate for people with hip/knee OA when done correctly"

- ◯ 0 = Not at all appropriate for people with hip/knee OA
- ◯ 1
- ◯ 2
- ◯ 3
- ◯ 4
- ◯ 5
- ◯ 6
- ◯ 7
- ◯ 8
- ◯ 9
- ◯ 10 = Completely appropriate for people with hip/knee OA when done correctly

Is this movement safe to do unsupervised at home when done correctly?
Please select the one number on the scale that best represents your opinion with 0 = "Not at all safe for people to perform at home unsupervised" and 10= "Completely safe for people to perform at home unsupervised when done correctly"

- ◯ 0 = Not at all safe for people to perform at home unsupervised
- ◯ 1
- ◯ 2
- ◯ 3
- ◯ 4
- ◯ 5
- ◯ 6
- ◯ 7
- ◯ 8
- ◯ 9
- ◯ 10 = Completely safe for people to perform at home unsupervised when done correctly

Is this movement practical to be delivered online using pre-recorded videos that someone will watch at home?

For example: the movement would not be practical if it involved movements that require people to turn their back to the computer screen for long periods of time, making it hard to view the instructor/video, and/or if the movements required too much room space

- ◯ Yes
- ◯ No

Section 29

Movement 21 Turn Body, Deflect, Parry and Punch

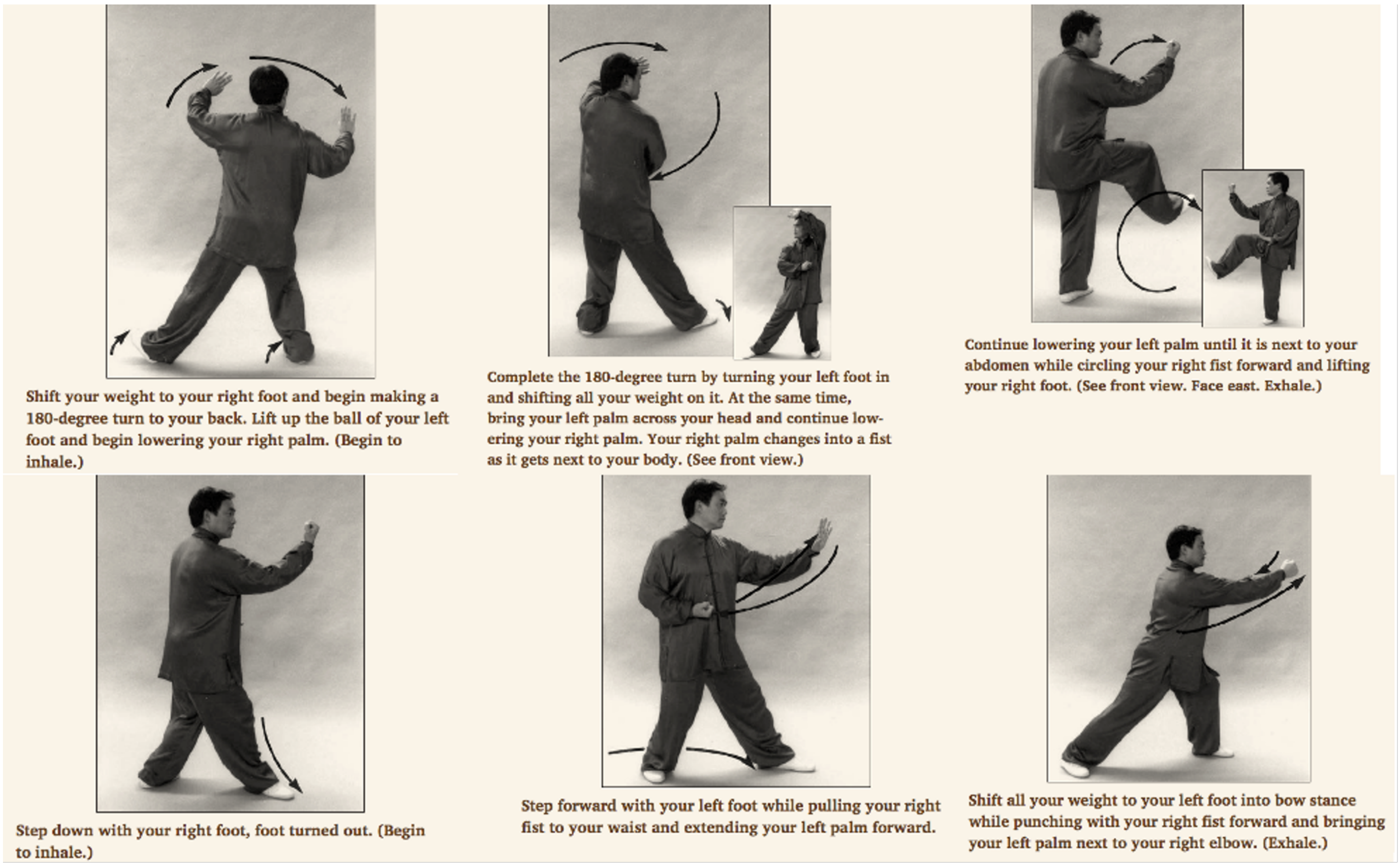

Shift your weight to your right foot and begin making a 180-degree turn to your back. Lift up the ball of your left foot and begin lowering your right palm. (Begin to inhale.)

Complete the 180-degree turn by turning your left foot in and shifting all your weight on it. At the same time, bring your left palm across your head and continue lowering your right palm. Your right palm changes into a fist as it gets next to your body. (See front view.)

Continue lowering your left palm until it is next to your abdomen while circling your right fist forward and lifting your right foot. (See front view. Face east. Exhale.)

Step down with your right foot, foot turned out. (Begin to inhale.)

Step forward with your left foot while pulling your right fist to your waist and extending your left palm forward.

Shift all your weight to your left foot into bow stance while punching with your right fist forward and bringing your left palm next to your right elbow. (Exhale.)

Is this movement appropriate for people with knee/hip osteoarthritis when done correctly?
Please select the one number on the scale that best represents your opinion with 0 = "Not at all appropriate for people with hip/knee OA" and 10 = "Completely appropriate for people with hip/knee OA when done correctly"

- ◯ 0 = Not at all appropriate for people with hip/knee OA
- ◯ 1
- ◯ 2
- ◯ 3
- ◯ 4
- ◯ 5
- ◯ 6
- ◯ 7
- ◯ 8
- ◯ 9
- ◯ 10 = Completely appropriate for people with hip/knee OA when done correctly

Is this movement safe to do unsupervised at home when done correctly?
Please select the one number on the scale that best represents your opinion with 0 = "Not at all safe for people to perform at home unsupervised" and 10= "Completely safe for people to perform at home unsupervised when done correctly"

- ◯ 0 = Not at all safe for people to perform at home unsupervised
- ◯ 1
- ◯ 2
- ◯ 3
- ◯ 4
- ◯ 5
- ◯ 6
- ◯ 7
- ◯ 8
- ◯ 9
- ◯ 10 = Completely safe for people to perform at home unsupervised when done correctly

Is this movement practical to be delivered online using pre-recorded videos that someone will watch at home?

For example: the movement would not be practical if it involved movements that require people to turn their back to the computer screen for long periods of time, making it hard to view the instructor/video, and/or if the movements required too much room space

◯ Yes
◯ No

Section 30

Movement 21 Modified- Deflect, Parry and Punch

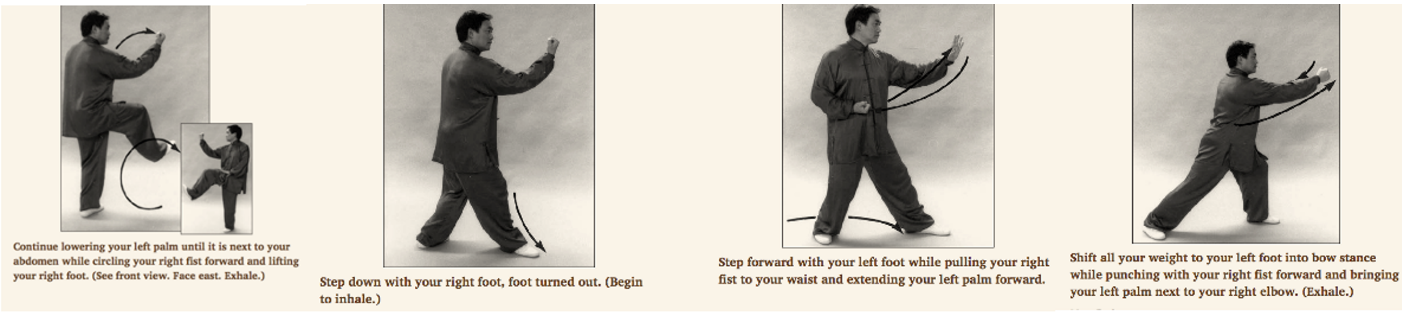

Is this movement appropriate for people with knee/hip osteoarthritis when done correctly?
Please select the one number on the scale that best represents your opinion with 0 = "Not at all appropriate for people with hip/knee OA" and 10 = "Completely appropriate for people with hip/knee OA when done correctly"

- ◯ 0 = Not at all appropriate for people with hip/knee OA
- ◯ 1
- ◯ 2
- ◯ 3
- ◯ 4
- ◯ 5
- ◯ 6
- ◯ 7
- ◯ 8
- ◯ 9
- ◯ 10 = Completely appropriate for people with hip/knee OA when done correctly

---

Is this movement safe to do unsupervised at home when done correctly?
Please select the one number on the scale that best represents your opinion with 0 = "Not at all safe for people to perform at home unsupervised" and 10= "Completely safe for people to perform at home unsupervised when done correctly"

- ◯ 0 = Not at all safe for people to perform at home unsupervised
- ◯ 1
- ◯ 2
- ◯ 3
- ◯ 4
- ◯ 5
- ◯ 6
- ◯ 7
- ◯ 8
- ◯ 9
- ◯ 10 = Completely safe for people to perform at home unsupervised when done correctly

---

Is this movement practical to be delivered online using pre-recorded videos that someone will watch at home?

For example: the movement would not be practical if it involved movements that require people to turn their back to the computer screen for long periods of time, making it hard to view the instructor/video, and/or if the movements required too much room space

- ◯ Yes
- ◯ No

Section 31

Movement 22 Apparent Closing a Door

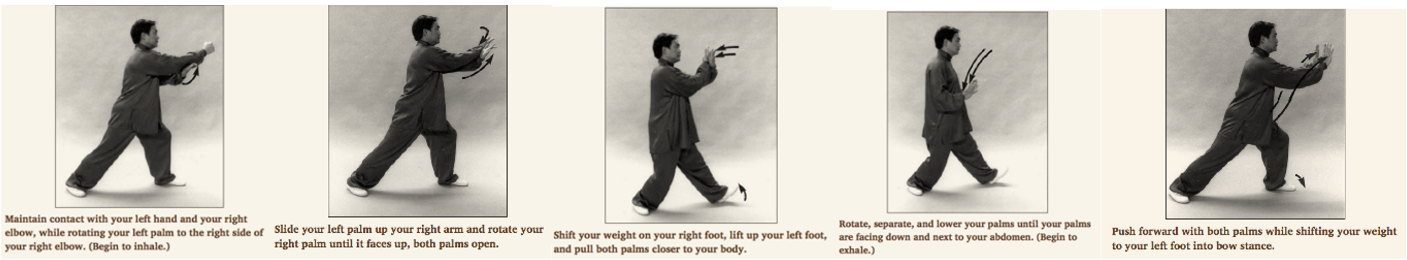

Is this movement appropriate for people with knee/hip osteoarthritis when done correctly?
Please select the one number on the scale that best represents your opinion with 0 = "Not at all appropriate for people with hip/knee OA" and 10 = "Completely appropriate for people with hip/knee OA when done correctly"

- ◯ 0 = Not at all appropriate for people with hip/knee OA
- ◯ 1
- ◯ 2
- ◯ 3
- ◯ 4
- ◯ 5
- ◯ 6
- ◯ 7
- ◯ 8
- ◯ 9
- ◯ 10 = Completely appropriate for people with hip/knee OA when done correctly

---

Is this movement safe to do unsupervised at home when done correctly?
Please select the one number on the scale that best represents your opinion with 0 = "Not at all safe for people to perform at home unsupervised" and 10= "Completely safe for people to perform at home unsupervised when done correctly"

- ◯ 0 = Not at all safe for people to perform at home unsupervised
- ◯ 1
- ◯ 2
- ◯ 3
- ◯ 4
- ◯ 5
- ◯ 6
- ◯ 7
- ◯ 8
- ◯ 9
- ◯ 10 = Completely safe for people to perform at home unsupervised when done correctly

---

Is this movement practical to be delivered online using pre-recorded videos that someone will watch at home?

For example: the movement would not be practical if it involved movements that require people to turn their back to the computer screen for long periods of time, making it hard to view the instructor/video, and/or if the movements required too much room space

- ◯ Yes
- ◯ No

Section 32

Movement 23 Cross Hands

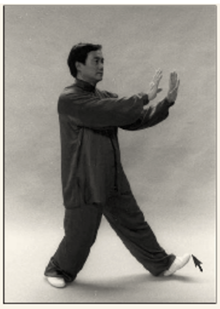

Shift your weight to your right foot, lift up the ball of your left foot, and begin turning your body 180 degrees to your right. (Begin to inhale.)

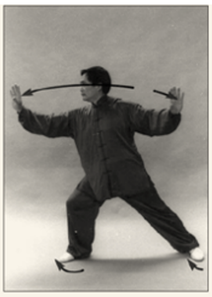

Turn your left foot in until it points forward. Then turn your right foot out while you rotate and extend your right palm to your right. Pull your left palm to your left slightly.

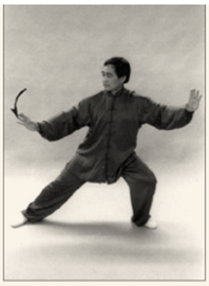

Shift your weight to your left foot and begin lowering your palms. (Begin to exhale.)

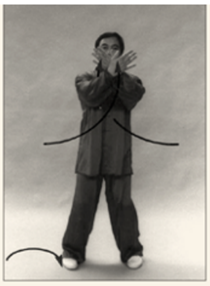

Bring your right foot closer to your left until both feet are shoulder width apart while scooping both palms down and up. Cross wrists in front of your face with the right palm on the outside. Stand up slightly, but keep your knees bent. (Face south.)

Is this movement appropriate for people with knee/hip osteoarthritis when done correctly?
Please select the one number on the scale that best represents your opinion with 0 = "Not at all appropriate for people with hip/knee OA" and 10 = "Completely appropriate for people with hip/knee OA when done correctly"

- ◯ 0 = Not at all appropriate for people with hip/knee OA
- ◯ 1
- ◯ 2
- ◯ 3
- ◯ 4
- ◯ 5
- ◯ 6
- ◯ 7
- ◯ 8
- ◯ 9
- ◯ 10 = Completely appropriate for people with hip/knee OA when done correctly

---

Is this movement safe to do unsupervised at home when done correctly?
Please select the one number on the scale that best represents your opinion with 0 = "Not at all safe for people to perform at home unsupervised" and 10= "Completely safe for people to perform at home unsupervised when done correctly"

- ◯ 0 = Not at all safe for people to perform at home unsupervised
- ◯ 1
- ◯ 2
- ◯ 3
- ◯ 4
- ◯ 5
- ◯ 6
- ◯ 7
- ◯ 8
- ◯ 9
- ◯ 10 = Completely safe for people to perform at home unsupervised when done correctly

---

Is this movement practical to be delivered online using pre-recorded videos that someone will watch at home?

For example: the movement would not be practical if it involved movements that require people to turn their back to the computer screen for long periods of time, making it hard to view the instructor/video, and/or if the movements required too much room space

- ◯ Yes
- ◯ No

Section 33

Movement 24 Closing Posture

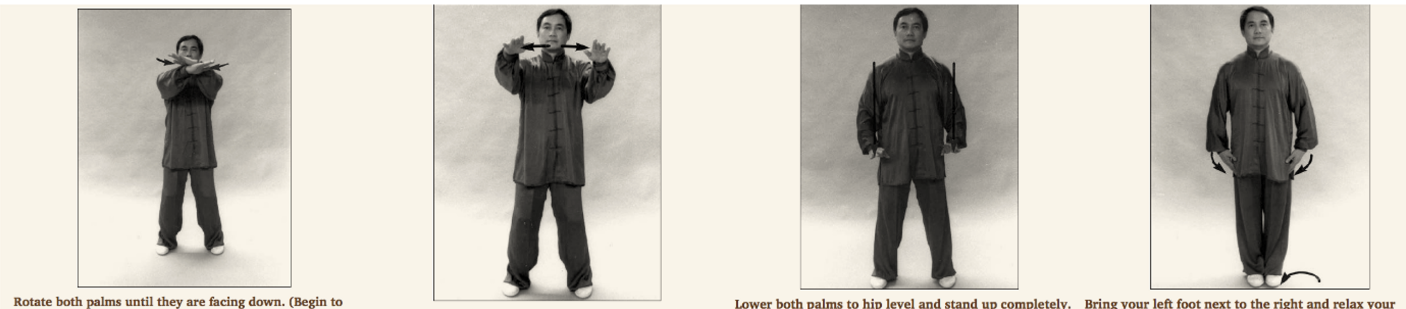

Rotate both palms until they are facing down. (Begin to inhale.)
Separate both palms until they are shoulder width apart.
Lower both palms to hip level and stand up completely. (Exhale.)
Bring your left foot next to the right and relax your hands down to your sides.

Is this movement appropriate for people with knee/hip osteoarthritis when done correctly?
Please select the one number on the scale that best represents your opinion with 0 = "Not at all appropriate for people with hip/knee OA" and 10 = "Completely appropriate for people with hip/knee OA when done correctly"

- ◯ 0 = Not at all appropriate for people with hip/knee OA
- ◯ 1
- ◯ 2
- ◯ 3
- ◯ 4
- ◯ 5
- ◯ 6
- ◯ 7
- ◯ 8
- ◯ 9
- ◯ 10 = Completely appropriate for people with hip/knee OA when done correctly

---

Is this movement safe to do unsupervised at home when done correctly?
Please select the one number on the scale that best represents your opinion with 0 = "Not at all safe for people to perform at home unsupervised" and 10= "Completely safe for people to perform at home unsupervised when done correctly"

- ◯ 0 = Not at all safe for people to perform at home unsupervised
- ◯ 1
- ◯ 2
- ◯ 3
- ◯ 4
- ◯ 5
- ◯ 6
- ◯ 7
- ◯ 8
- ◯ 9
- ◯ 10 = Completely safe for people to perform at home unsupervised when done correctly

---

Is this movement practical to be delivered online using pre-recorded videos that someone will watch at home?

For example: the movement would not be practical if it involved movements that require people to turn their back to the computer screen for long periods of time, making it hard to view the instructor/video, and/or if the movements required too much room space

- ◯ Yes
- ◯ No

Questions about Tai Chi exercise prescription:

The following questions will ask your opinion regarding how long each pre-recorded Tai Chi session should be and the weekly volume and frequency of Tai chi practice that you would recommend.

How long should each pre-recorded video session of Tai Chi movements go for?

◯ 20min ◯ 30min ◯ 40min ◯ 50min ◯ 60min

What volume (total min/week) of weekly Tai Chi practice would you recommend? (e.g., 120 minutes each week)

____

Each week, how frequently (days/week) should the participant perform the Tai Chi movements?

◯ 1 ◯ 2 ◯ 3 ◯ 4 ◯ 5 ◯ 6 ◯ 7

After the participants finish the 12-week unsupervised online Tai Chi program, what would you recommend them to practice at home afterwards?

Do you have any suggestions on the Tai Chi exercise prescription and/or comments on any parts of the survey?

Thank you [first_name_consent], for taking the survey.

We look forward to working with you to develop a program that is appropriate for people with hip/knee osteoarthritis, safe to do unsupervised and practical to be delivered online.

Please press submit to register your responses.
