# Supplementary material for: Development of a 12-Week Unsupervised Online Tai Chi Program for People With Hip and Knee Osteoarthritis: Mixed Methods Study
Source: JMIR Aging. 2024 Sep 30;7:e55322. doi: 10.2196/55322 (PMC11474117; doi:10.2196/55322)
Supplement: Multimedia Appendix 2 [file aging_v7i1e55322_app2.docx]

## **Multimedia Appendix 2. The 9 movements excluded from Survey 1**

|  | Movement names | |
| --- | --- | --- |
| 1 | Movement 11 | Single Whip |
| 2 | Movement 13 | Right Heel Kick |
| 3 | Movement 15 | Turn and Left Heel Kick |
| 4 | Movement 16a | Left Lower Body/ Snake Creeps Down / Single Whip Squatting Down |
| 5 | Movement 16a | Modified Left Lower Body/ Snake Creeps Down / Single Whip Squatting Down |
| 6 | Movement 16b | Golden Rooster /Stands on One Leg |
| 7 | Movement 17a | Right Lower Body/ Snake Creeps Down/ Single Whip Squatting Down |
| 8 | Movement 17b | Golden Rooster /Stands on One Leg |
| 9 | Movement 21 | Turn Body, Deflect, Parry and Punch |
