# Supplementary material for: Development of a 12-Week Unsupervised Online Tai Chi Program for People With Hip and Knee Osteoarthritis: Mixed Methods Study
Source: JMIR Aging. 2024 Sep 30;7:e55322. doi: 10.2196/55322 (PMC11474117; doi:10.2196/55322)
Supplement: Multimedia Appendix 3 [file aging_v7i1e55322_app3.docx]

## **Multimedia Appendix 3. The ranked list of Tai Chi movements based on Survey 2 (n=27)**

| **Tai Chi movement rank based on mean value** | **Movement Name** |
| --- | --- |
| 1 | Wave Hands Like Clouds |
| 2 | Commencing Posture |
| 3 | The Wild Horse Parts Its Mane, L&R |
| 4 | Brush knee and step forward, L&R |
| 5 | Fair Lady Works with Shuttles, L&R |
| 6 | Modified - Golden Rooster, R |
| 7 | Modified - Golden Rooster, L |
| 8 | Modified- Heel Kick, L |
| 9 | Apparent Closing a Door |
| 10 | Grasp Sparrow's Tail, L |
| 11 | Modified- Heel Kick, R |
| 12 | White Crane Spreads Its Wings |
| 13 | Repulse Monkey, L&R |
| 14 | Grasp Sparrow's Tail, R |
| 15 | Playing the Lute |
| 16 | Movement 24 Closing Posture |
| 17 | Single Whip |
| 18 | High Pat on Horse |
| 19 | Fan Through Back/ Flash Arms |
| 20 | Cross Hands |
| 21 | Strike to the Ears with Both Fists |
| 22 | Needle at Sea Bottom |
| 23 | Modified- Deflect, Parry and Punch |
| 24 | Modified- Right Lower Body |
