# Supplementary material for: Development of a 12-Week Unsupervised Online Tai Chi Program for People With Hip and Knee Osteoarthritis: Mixed Methods Study
Source: JMIR Aging. 2024 Sep 30;7:e55322. doi: 10.2196/55322 (PMC11474117; doi:10.2196/55322)
Supplement: Multimedia Appendix 4 [file aging_v7i1e55322_app4.docx]

| **Tai Chi movements**   - Modify the 90-degree turn movement. Initiate with a 45-degree turn and progress to 90 degrees. Ensure even distribution of body weight throughout. - Incorporate a range of options for the single-leg stance movement to accommodate users at varying skill levels. - Modify other Tai Chi movements to ensure suitability for people with OA and little prior Tai Chi experience. - Consider adding a chair option for those who require additional support. |
| --- |
| **Tai Chi exercise prescription and program structure:**   - Consider starting with 30 minutes and gradually progressing to 40 minutes per session as more movements are introduced. - Confirmation that 12 week is an acceptable duration for the online program. - Integrate Qigong exercises as warm-up and cool-down routines. |
| **Martial application**   - Provide an explanation of the martial application to enhance understanding of the underlying principles behind each Tai Chi movement. |
| **Practicalities of recording the online Tai Chi program**   - Ensure both front and back views are recorded to provide comprehensive visual guidance. - Use mirror image for front view for movement demonstrations. - Integrate clear modification cues throughout for users with various abilities |
| **Recommandations post online Tai Chi program**   - Encourage continued practice using pre-recorded Tai Chi videos and increasing the frequency of Tai Chi sessions. - Encourage users to memorize the Tai Chi sequence and practice without relying on the video. - Recommend attending in-person Tai Chi classes |
| **Resources to be provided on the website**   - Provide links to external Tai Chi classes to facilitate easy access to instructors and schools - Provide still images illustrating Tai Chi movement basics with a printable movement sequence |
