# Supplementary material for: Development of a 12-Week Unsupervised Online Tai Chi Program for People With Hip and Knee Osteoarthritis: Mixed Methods Study
Source: JMIR Aging. 2024 Sep 30;7:e55322. doi: 10.2196/55322 (PMC11474117; doi:10.2196/55322)
Supplement: Multimedia Appendix 5 [file aging_v7i1e55322_app5.docx]

## **Multimedia Appendix 5. “My Joint Tai Chi” website section description**

| **About Tai Chi**  This section provides general written information about Tai Chi and Qigong, health benefit of Tai Chi and Qigong, and its movement principle. |
| --- |
| **My Joint Tai Chi Program**  This section contains the self-directed 12-week progressive Tai Chi program (“My Joint Tai Chi” program) guided by an instructor delivering the program in a series of 12 pre-recorded videos (one video per week). In the Tai Chi program, each video (40-45 mins) includes a warm-up consisting of an introduction by the instructor, standing meditation, breathing exercises, and Qigong intended to warm up the body. The central portion of each video uses a modified 10-form Yang style Tai Chi which involves slow and controlled movements. Modifications to the movements were made to ensure suitability for people with lower limb OA and little prior Tai Chi experience. Each video ends with a cool-down consisting of breathing practices and relaxation exercises. The 12-week program starts with simple Tai Chi movements with new movements are added each week to progress the program. Program users can undertake the program in their home at a time of their choosing. Users are instructed to complete each week’s Tai Chi session 3 times per week and continue for a 12-week period. This section also provides an introduction to the Tai Chi program, how to prepare, information on managing exercise pain, optional resources including a skill video and a printable Tai Chi movement sequence, and recommendations after people finish the recommended 12-week program. |
| **Tai Chi Exercise adherence support app – My Exercise Messages**  The “My Exercises Messages” app was specifically designed for people with hip and/or knee OA. The My Joint Tai Chi program recommends users download and commence using the app in week 1 of their Tai Chi program and are encourages to use it for the full duration of the program (12 weeks). The My Exercise Messages app is free to download from the App Store (Apple devices) and Google Play Store (Android devices). After downloading the app, the app will prompt users to input i) their name; ii) the duration of the exercise program (12 weeks); and iii) the number of days per week they are aiming to complete their Tai Chi exercise (3 days per week). Seven days after setting up the app (and each week thereafter), they will receive a smartphone notification, from the app, prompting them to enter the app and record the number of days they completed their recommended Tai Chi exercise, in the past week. If they report less than three days in the past week (low adherence), they will then be prompted to select a reason for low adherence (from a pre-specified list of common exercise barriers for people with OA). Users will then receive a tailored message containing a behaviour change technique suggestion to help them overcome their identified exercise barrier in the coming week. Users also receive 2 notifications each week containing messages designed to facilitate regular exercise participation. |
| **About Knee Osteoarthritis**  This section contains written information about living with knee OA, knee OA treatments, exercise as treatment for knee OA. Written information is supported by video interviews of people with knee OA and OA experts. Participants can view the written and audio-visual material while doing the Tai Chi exercise program prescribed within the ‘My Tai Chi program’ section of the website. |
