# Supplementary material for: Development of a 12-Week Unsupervised Online Tai Chi Program for People With Hip and Knee Osteoarthritis: Mixed Methods Study
Source: JMIR Aging. 2024 Sep 30;7:e55322. doi: 10.2196/55322 (PMC11474117; doi:10.2196/55322)
Supplement: Multimedia Appendix 6 [file aging_v7i1e55322_app6.pdf]

Modified Yang Style 10 Form Sequence

MYJOINT TAI CHI

1. Commencement

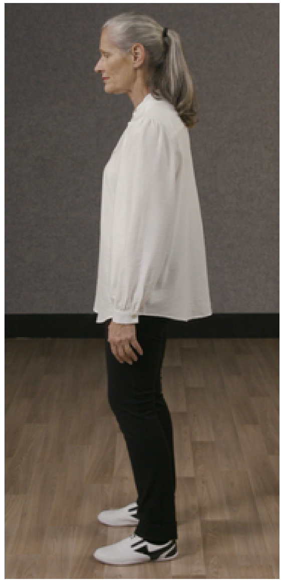

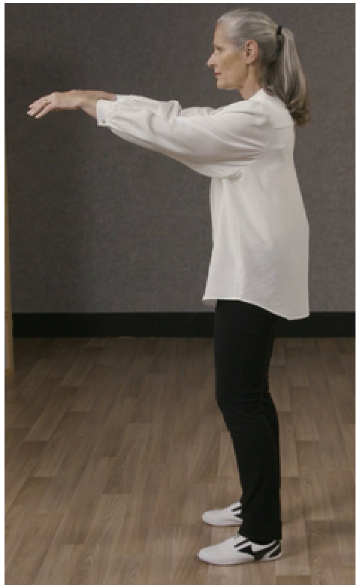

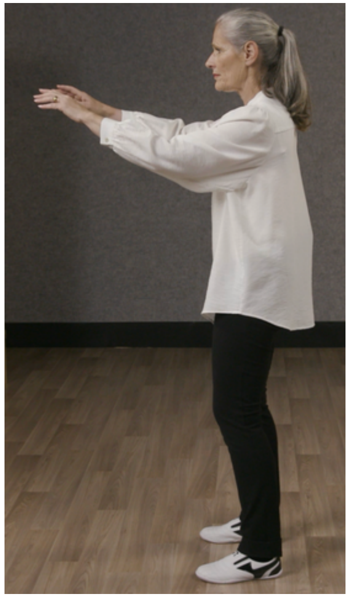

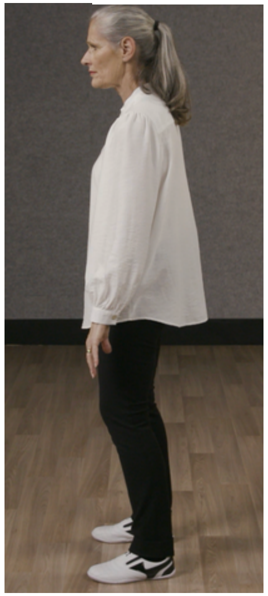

2. Repulse Monkey

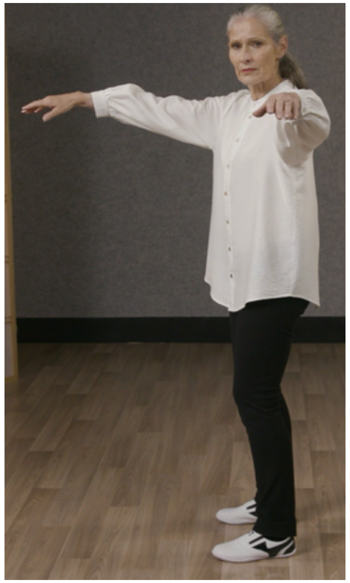

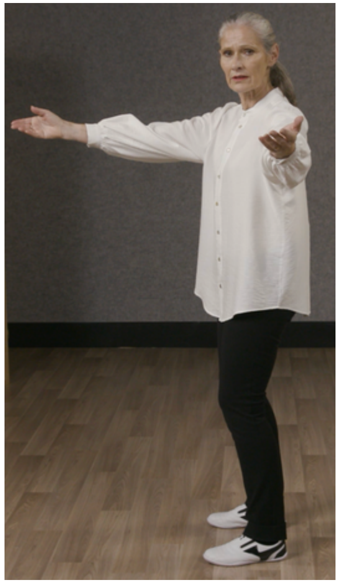

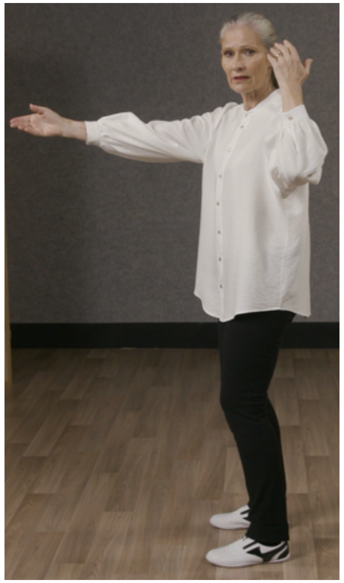

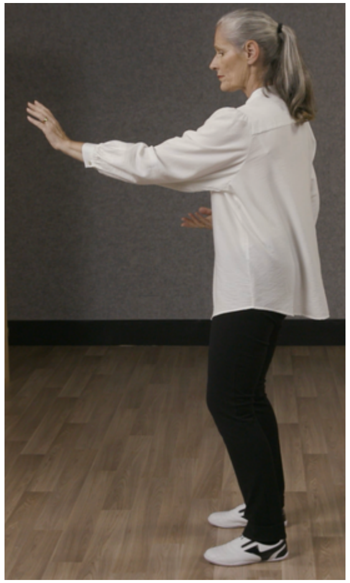

POSTERA CRESCAM LAUDE
THE UNIVERSITY OF MELBOURNE

3. Brush Knee

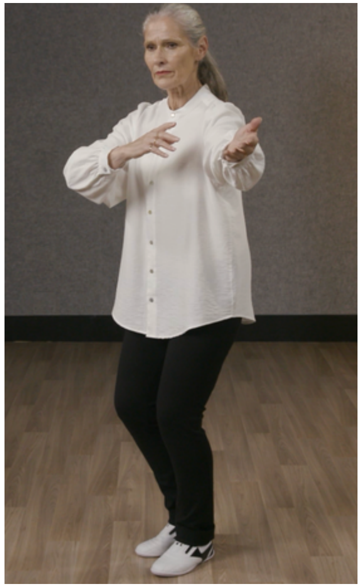

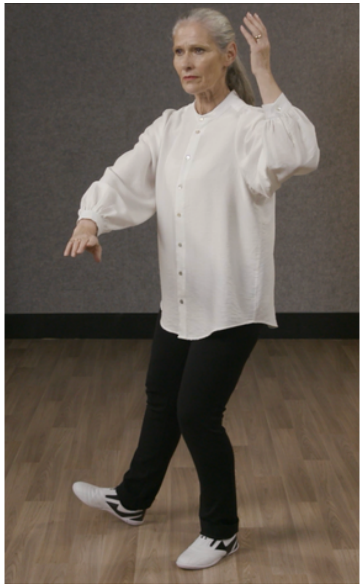

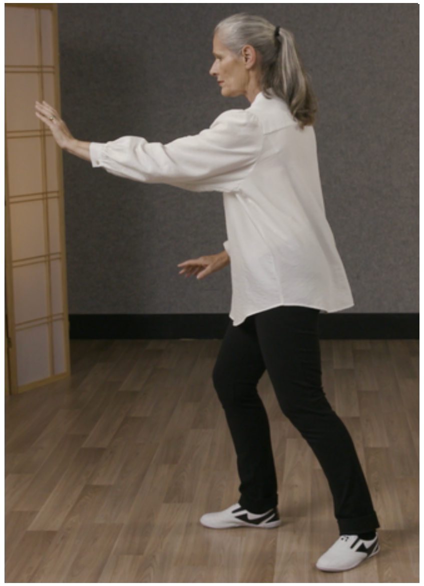

4. Part the Wild Horse's Mane

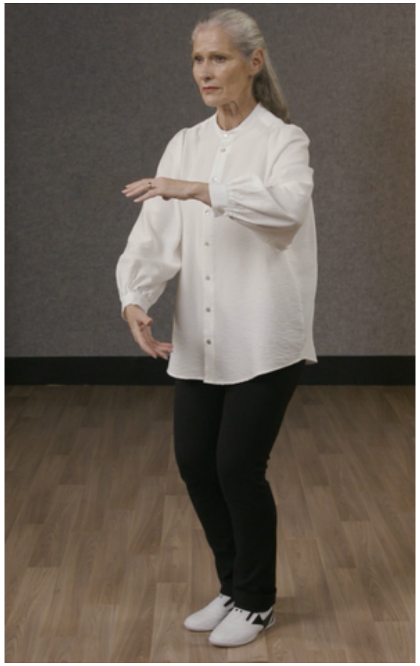

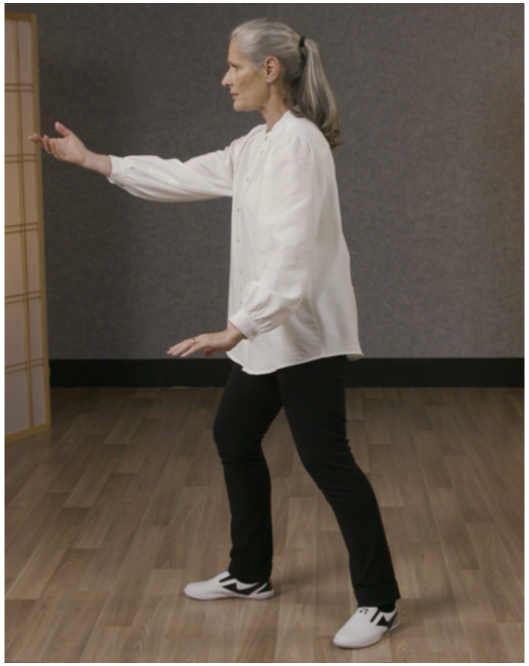

THE UNIVERSITY OF MELBOURNE

5. Cloud Hands

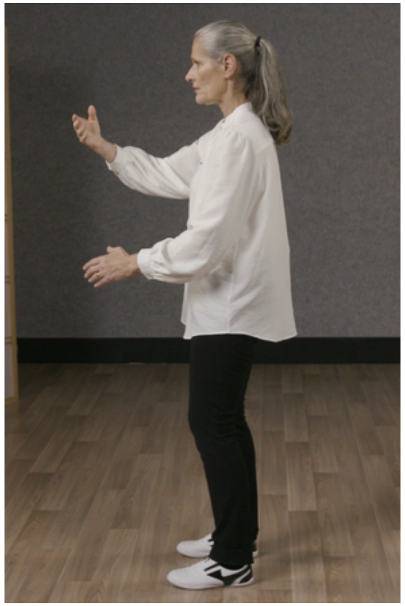

6. Golden Rooster Stands on One Leg

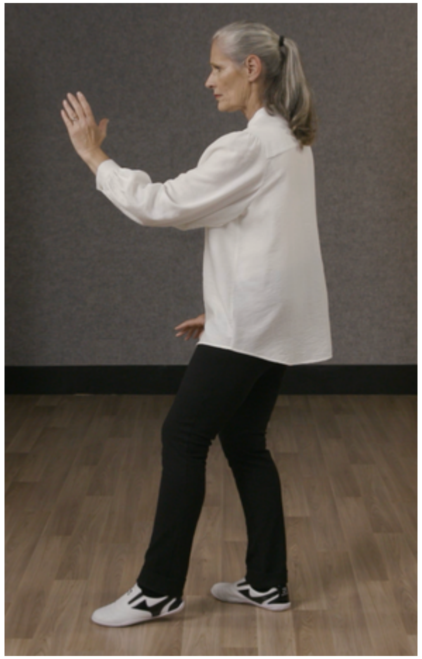

Easier Option

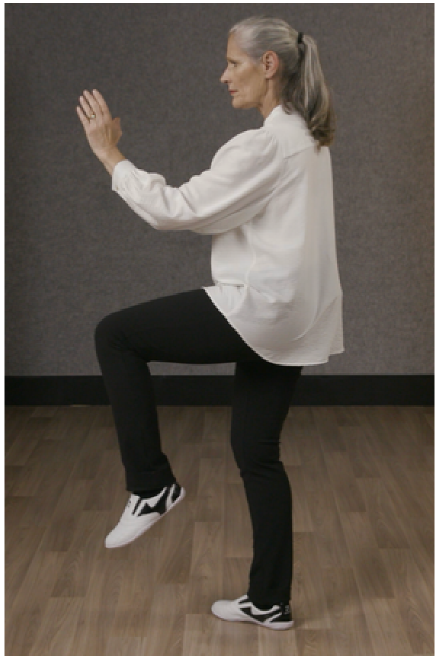

Harder Option

THE UNIVERSITY OF MELBOURNE

MYJOINT TAI CHI

7. Kick with Heel

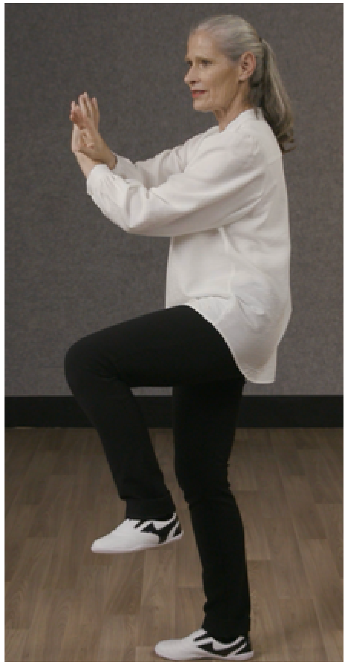

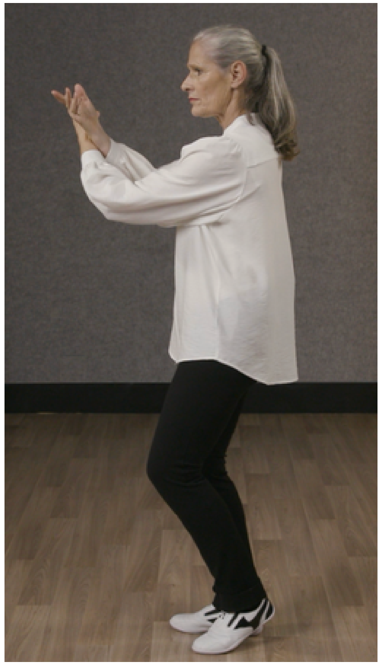

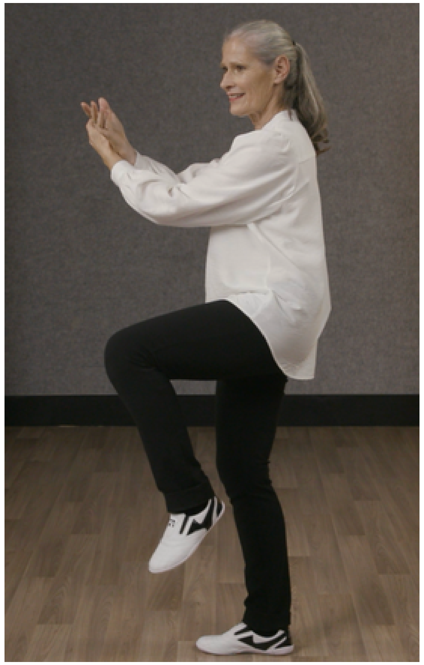

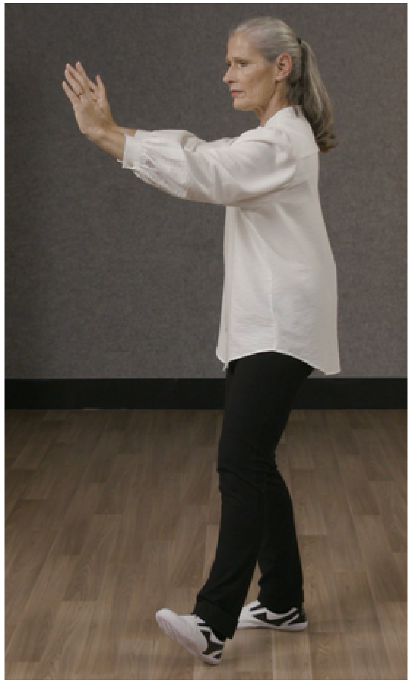

Easier Option

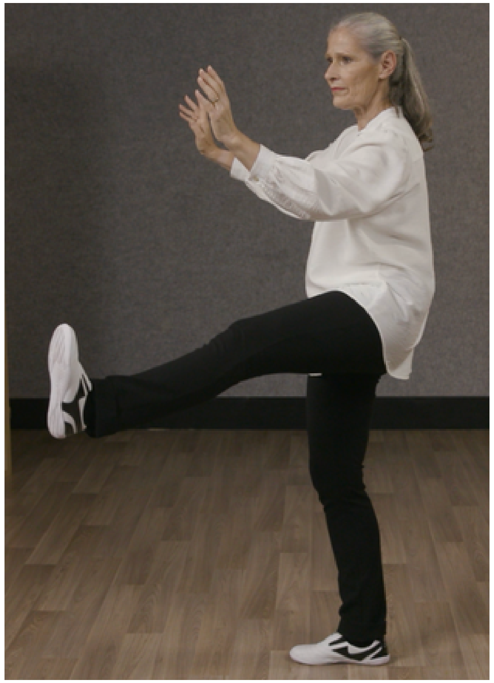

Harder Option

POSTERA CRESCAM LAUDE
THE UNIVERSITY OF MELBOURNE

8. Stroke Peacock's Tail/ Grasp Bird's Tail

MYJOINT TAI CHI

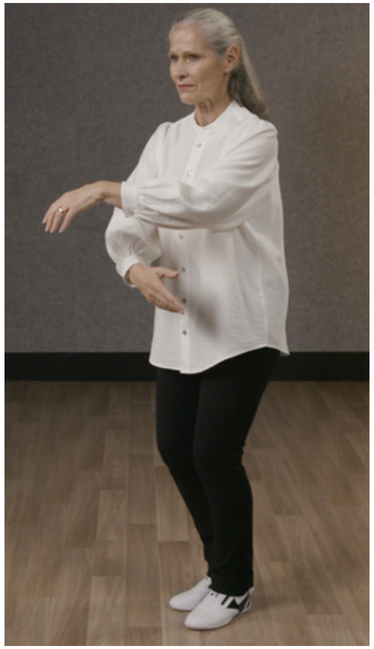

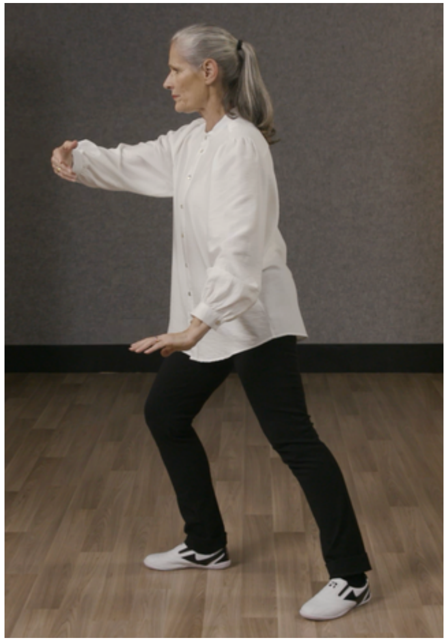

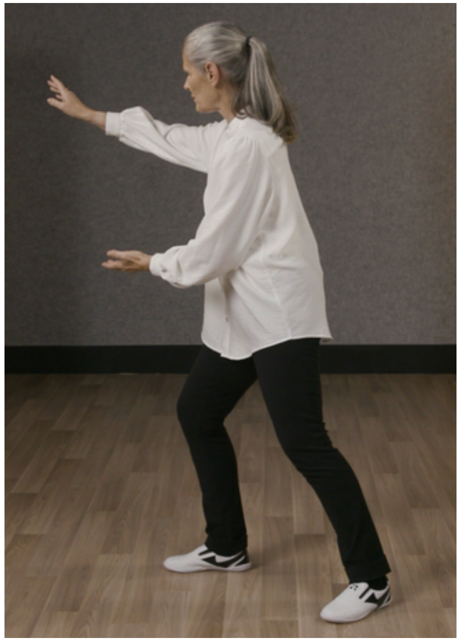

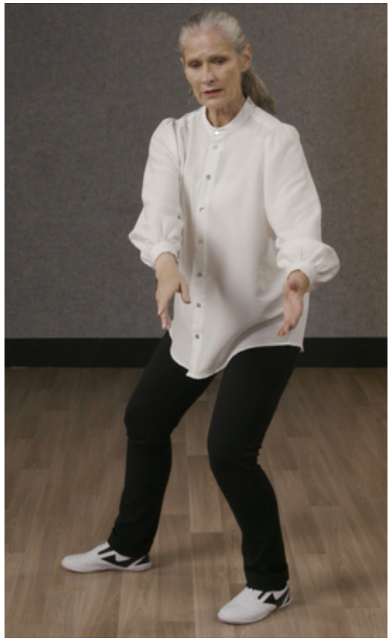

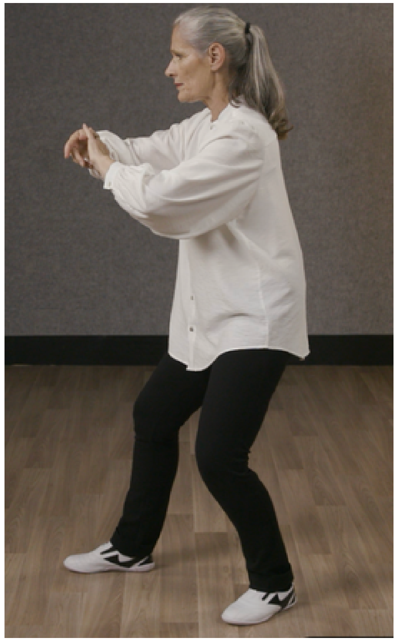

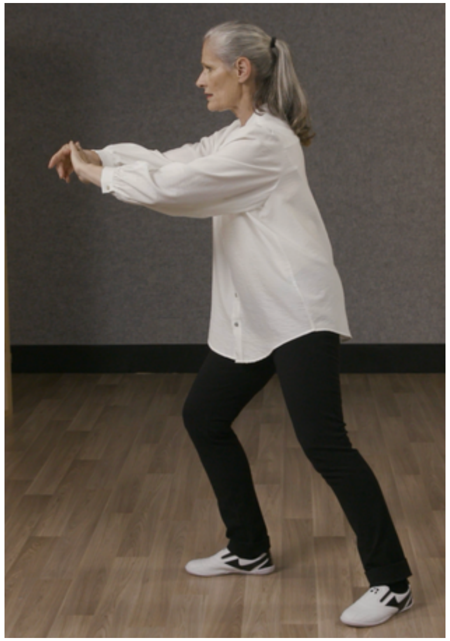

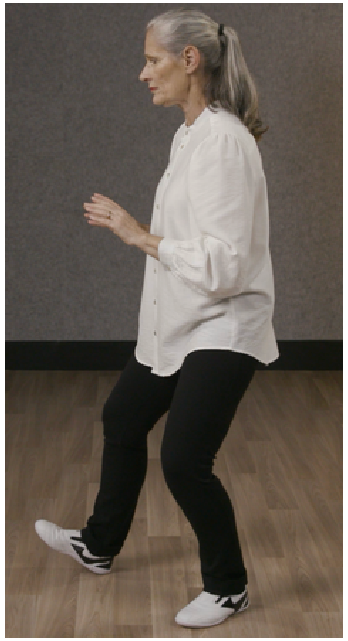

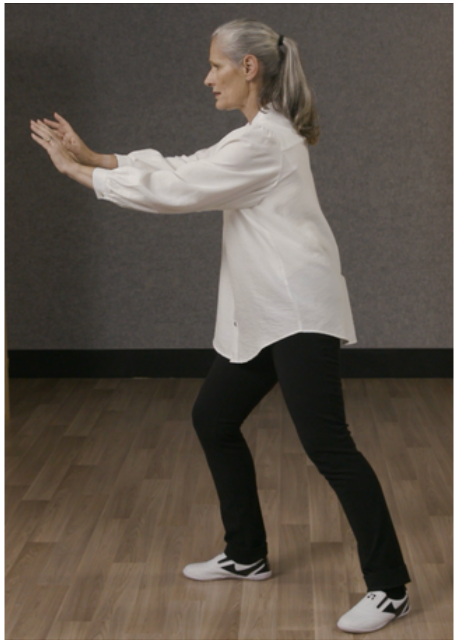

POSTERA CRESCAM LAUDE
THE UNIVERSITY OF MELBOURNE

MYJOINT TAI CHI

9. Embrace the Tiger

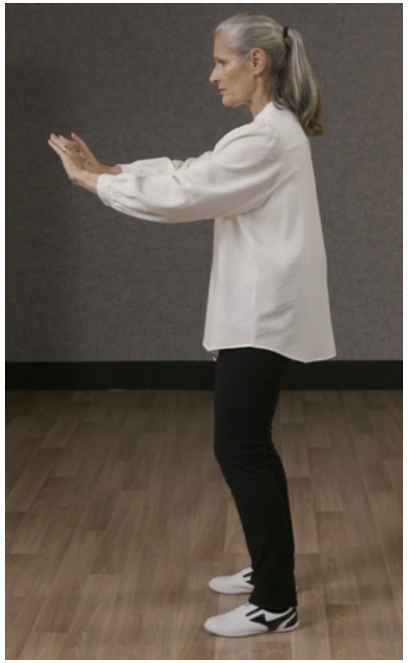

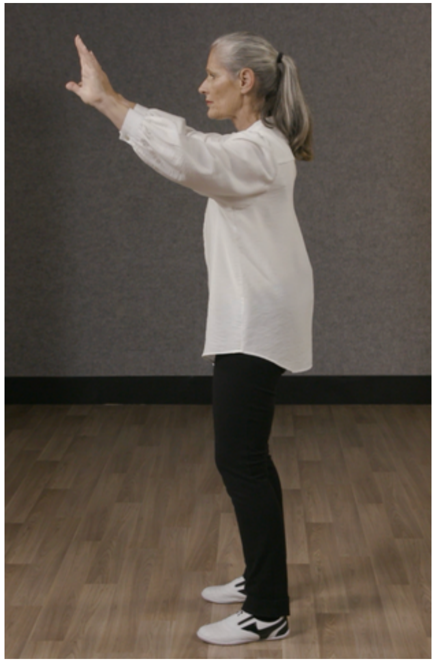

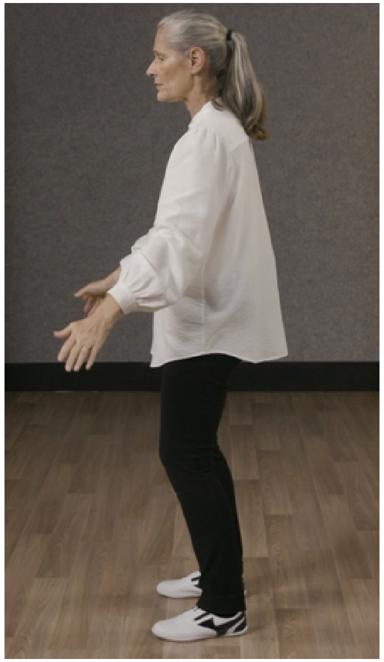

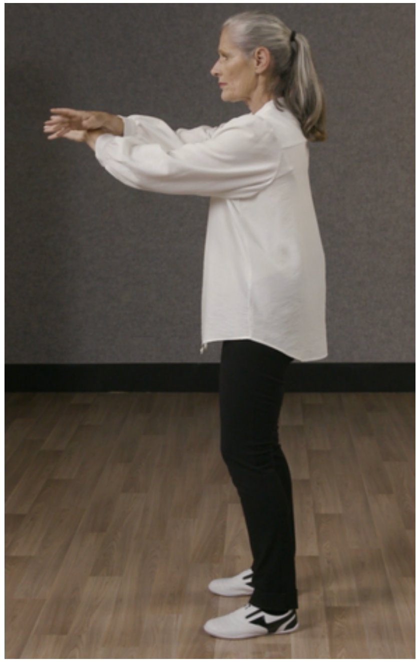

10. Closing

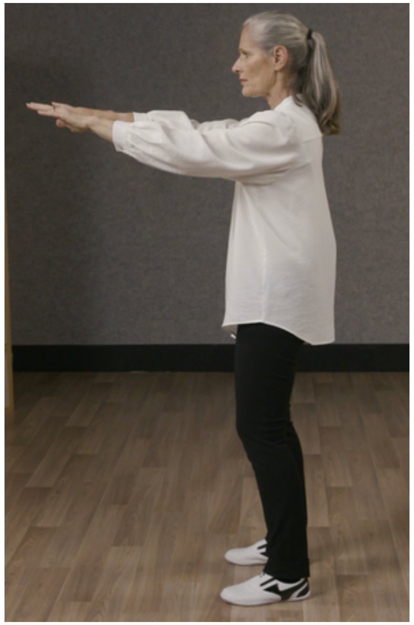

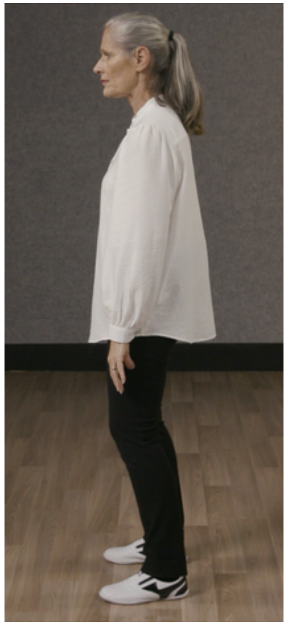

THE UNIVERSITY OF MELBOURNE
