# Supplementary material for: Development of a 12-Week Unsupervised Online Tai Chi Program for People With Hip and Knee Osteoarthritis: Mixed Methods Study
Source: JMIR Aging. 2024 Sep 30;7:e55322. doi: 10.2196/55322 (PMC11474117; doi:10.2196/55322)
Supplement: Multimedia Appendix 7 [file aging_v7i1e55322_app7.docx]

## **Multimedia Appendix 7. Usability issues identified and corresponding implemented solutions**

| **Usability issues** | **Solutions implemented in the final “My Tai Chi” website** |
| --- | --- |
| **User interface** |  |
| - Hyperlinks with lengthy sentence of instructions on the homepage was distracting. | - Removed the hyperlink and presented the instructions in concise bullet points. |
| - The font color (light grey) used for references at the end of each page was too faint and difficult to read. | - Changed the colour to dark blue to improve readability. |
| - Certain pages were text-heavy. | - Changed the subtitle color to orange and restructure the paragraph to be more digestible. Introduced additional images and small icons to make the page more visually engaging. |
| - The order of the section tabs was confusing. | - Reordered section tabs to align with a more intuitive reading flow. |
| **Tai Chi support app (“My Exercise Messages”)** | |
| - Users were uncertain whether the actual Tai Chi is in the app. | - Clarified the instruction on the page and emphasized that this app does “NOT” contain the actual Tai Chi program. |
| - Typing the app name in the Google Play/App Store was perceived as cumbersome | - Incorporated a direct hyperlink to the icons to make it easier for downloading. |
